# ERCAD: A Parametric Reactor Design Tool That Enables Rapid Prototyping and Optimization of Electrochemical Reactors through 3D Printing

**DOI:** 10.1021/acscentsci.4c00988

**Published:** 2024-09-27

**Authors:** David M. Heard, Sam W. Deeks, Alastair J. J. Lennox

**Affiliations:** School of Chemistry, University of Bristol, Cantock’s Close, Bristol, U.K., BS8 1TS

## Abstract

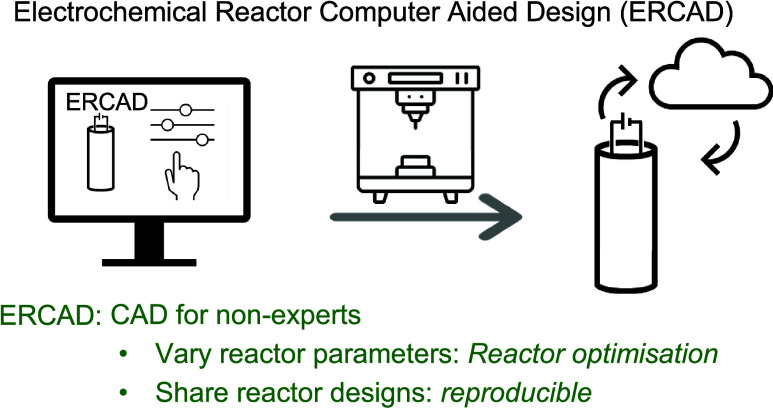

The reactors are an essential component of electrosynthetic reactions. As the electron transfer processes are heterogeneous, the reactors have a significant impact on reaction outcomes. This has resulted in reaction reproducibility being problematic, which commercial reactors alleviate somewhat but are expensive and cannot be optimized or iterated upon. Using 3D printing, rapid prototyping of bespoke reactors should facilitate investigation of the sensitivity of key reactor parameters, enable reactor optimization, and improved reproducibility through sharing of the print files. However, the bottleneck to this approach is the Computer Aided Design (CAD) of the reactors, which typically requires specialist knowledge and training to do. This has resulted in 3D printing not being typically used in the field of electrosynthesis. Herein, we showcase the development and application of a user-friendly, open-source software tool that can be used to produce Electrochemical Reactor CAD (ERCAD) designs simply and easily by nonexperts. We demonstrate its use to design and print reactors for the analysis, optimization, screening, and scaleup of electrosynthetic reactions. Using this parametric design tool, chemists with no design experience or skills can now easily create, print, test, and share their reactors.

## Abstract 2

Herein, we present the parametric electrochemical reactor design tool ERCAD, which enables nonexperts to produce CAD files to print, test, and share reactors, improving optimization and reproducibility in electrosynthesis.

## Introduction

Electrochemistry is a versatile technique to conduct redox reactions in synthesis,^1−3^ including in the fields of organic synthesis, biomass valorization, CO_2_ reduction, and biofuel energy conversion. The technique has shown benefits to synthetic transformations by achieving different or enhanced selectivity, sustainability, and safety compared to traditional approaches.^4−7^ These general benefits originate from several unique features of electrochemical reactions, including the ability to select any redox potential across the pair of electrodes, the spatial separation of the two half reactions, and the inherently heterogeneous nature of the electron-transfer between the electrode and the electrolyte solution.

Using an electrode to mediate heterogeneous electron-transfer creates an opportunity to deviate into different mechanisms. On the one extreme, the electrode surface is intimately involved in electron transfer and can act as a catalyst for this, and on the other extreme, it is inert and participates in outer-sphere electron transfer.^8,9^ In addition, the heterogeneous system creates a locally high concentration of reactive intermediate, which is a unique feature that necessarily enables certain reactions to occur, such as Kolbe dimerization.

**Figure 1:**
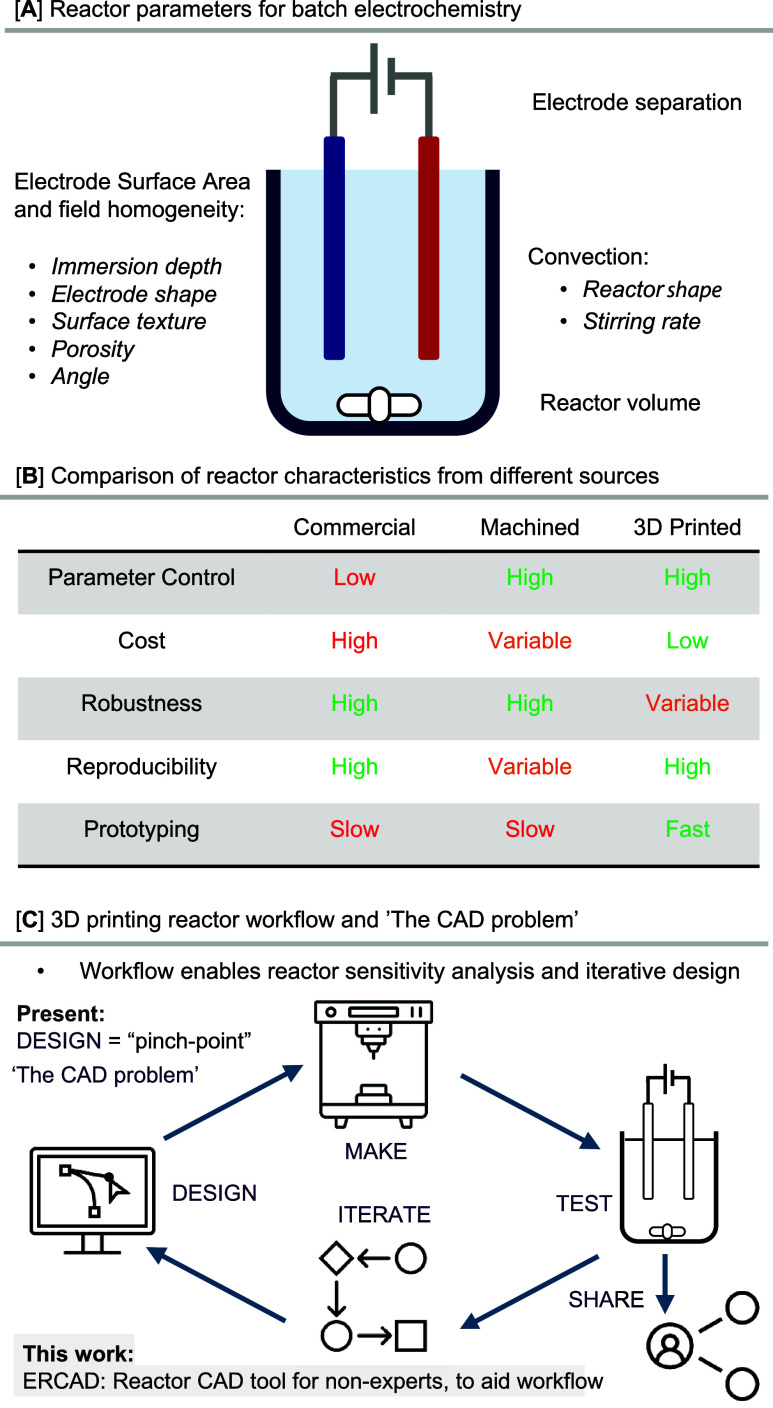
(A) Reactor parameters are important for electrochemical reactions. (B) Comparison of reactor sources. (C) Reactor optimization workflow. 3D printer icon downloaded from Vecteezy.com.

While the advantages in using electrodes for electron transfer are evident, they introduce complications and complexities into the reaction setups, or “reactors”, they are used in. These complications are especially apparent in the design and operation of batch electrolysis setups, which compare unfavorably to the simplicity of round-bottom flasks that are employed with conventional redox reagents.^10^ The heterogeneous reaction creates vessel-dependent effects on reaction outcomes, such as the yield and mass balance. These effects arise from the size, number, angle, and positioning of the electrodes,^8,11,12^ as well as the vessel shape and size, the efficiency of mixing through convection, and the electrode material. The interplay of these factors with reaction stirring and surface area:volume ratios, among other factors, must also be considered (Figure 1A).

To enable facile, reproducible reaction setups, several research groups and companies have reported elegant reactor designs, leading to the availability of several commercial solutions.^13−19^ However, commercial reactors or those machined by a skilled workshop, either in-house or externally, cannot be easily iterated upon without significant cost and lengthy lead times, which can make it difficult to investigate the sensitivity of, and optimize, reactor-based parameters. There is therefore space for an approach to reactor design, optimization, and dissemination that enables low-cost, rapid prototyping to allow reactors to be readily tailored to the reaction being studied, with the ability to vary important reactor parameters as necessary (Figure 1B).

The 3D printing of polypropylene (PP), which is inert to most organic solvents used in electrosynthesis, is emerging as a valuable, cost-effective approach to the design of bespoke reactors for organic synthesis^20−25^ (Figure 1B). This rapid and facile manufacturing technique has been shown to be applicable with enabling synthesis technologies, including in flow reactors,^26−30^ and photochemistry.^31−35^ The use of 3D printing for electrochemistry is comparatively much less explored,^36−38^ with the greatest attention on electroanalytical devices/sensors^39−41^ and energy-related applications,^37,42,43^ and with only very few examples in the field of electrosynthesis.^24,44−46^

A critical component in formulating custom reactors for 3D printing is Computer Aided Design (CAD), which is the construction of a three-dimensional digital design. While CAD is a powerful process, a distinct skillset is required that typically demands training to acquire and significant time and experience to master. This “CAD-problem” has therefore become a major pinch-point in the use of 3D printing in chemistry and has created a hurdle to adoption, especially to print reactors for electrochemistry that have additional degrees of complexity.^47−49^ We were drawn to address this problem after recognizing that many research groups use 3D printers to augment their laboratories,^50−52^ and the graphics file (.stl) for these various labware items are then made freely available.^53−55^ With an influx of low-cost 3D printers and print filaments on the market, we envisaged that if the “CAD-problem” could be overcome, this same trend could be useful in the field of electrosynthesis (Figure 1C). Hence, we proposed that an accessible design tool could be developed to rapidly output bespoke reactor designs with complete and facile control of all the key electrochemical parameters without having any prior skills or knowledge of CAD. In this way, the .stl files required for 3D printing can be rapidly produced and shared as necessary. Herein, we describe the development of such a design tool, electrochemical reactor computer-assisted design (ERCAD), for the facile creation and manipulation of electrochemical batch reactors.

## Results and Discussion

### Parametric Reactor Design Tool

While most Computer-Aided Design (CAD) software uses a graphic user interface in which a variety of tools are used to shape 3D objects, e.g., Fusion360, SolidWorks, TinkerCAD, these powerful packages require time or training to master. While they can be used to design chemical reactors,^22,24,33,56^ they do not intuitively lend themselves to such a task and an additional challenge is presented in easily iterating the designs. Alternatively, a code-based approach to CAD can be taken, which appears less accessible than graphical CAD packages but allows for a set of bespoke tools to be created. This makes iterations of the design straightforward, as only the necessary factors can be easily changed. With the aim to minimize the CAD knowledge required to design bespoke reactors, we adopted the code-based approach to be operated through a user-friendly customization tool.

We developed the reactor design tool ERCAD, using parametric design in the open-source CAD package OpenSCAD. Parametric design is an approach to CAD in which the key parameters of the final object are set by user-defined values. Changes in these values are then reflected in changes to the final object. Within ERCAD, this is implemented such that the variables to be modified are “chemistry-relevant” parameters. For example, rather than defining the height and diameter of a reactor, the size of the reactor is defined by a volume parameter. This parametric approach means that factors such as the interelectrode distance can be modified, thereby generating new reactor designs by changing a single value. This process allows reactor parameters to be easily varied and provides the opportunity to rationally study them through cycles of design-and-prototype.

**Figure 2:**
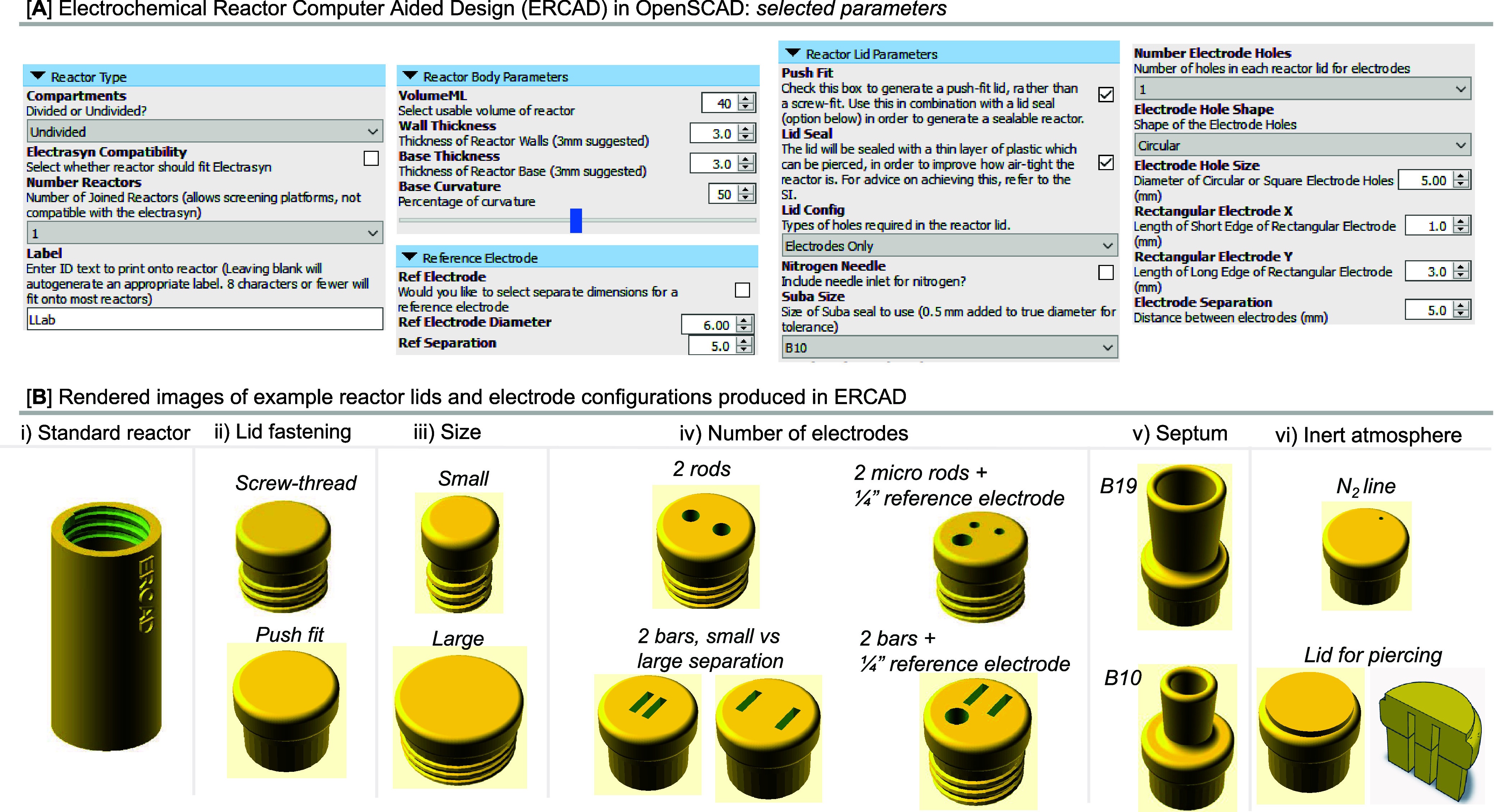
(A) The design process is achieved by entering chemistry-relevant values into a series of menus rather than requiring an in-depth understanding of CAD. (B) A variety of example lids are shown that can be produced from ERCAD.

ERCAD can be accessed easily by downloading OpenSCAD and importing the ERCAD tool. The reactor of choice is designed by inputting the desired parameters, of which up to 30 can be adjusted from drop-down lists or scales (Figure 2A). OpenSCAD can render an image of the reactor on screen, allowing the end-user to visually inspect the reactor and spot any design issues. The coding behind ERCAD can be viewed and edited through OpenSCAD, allowing it to be modified, for example, if imperial units are preferred, or extended to suit future users by those familiar with the OpenSCAD functional programming language. No restrictions are implemented on the reactor designs. While this freedom is required for full control, it comes at the cost of enabling the possibility to render impossible configurations. Reactor configurations designed through ERCAD can be saved and exported as an. stl file for printing or a. json file for further editing.

**Figure 3:**
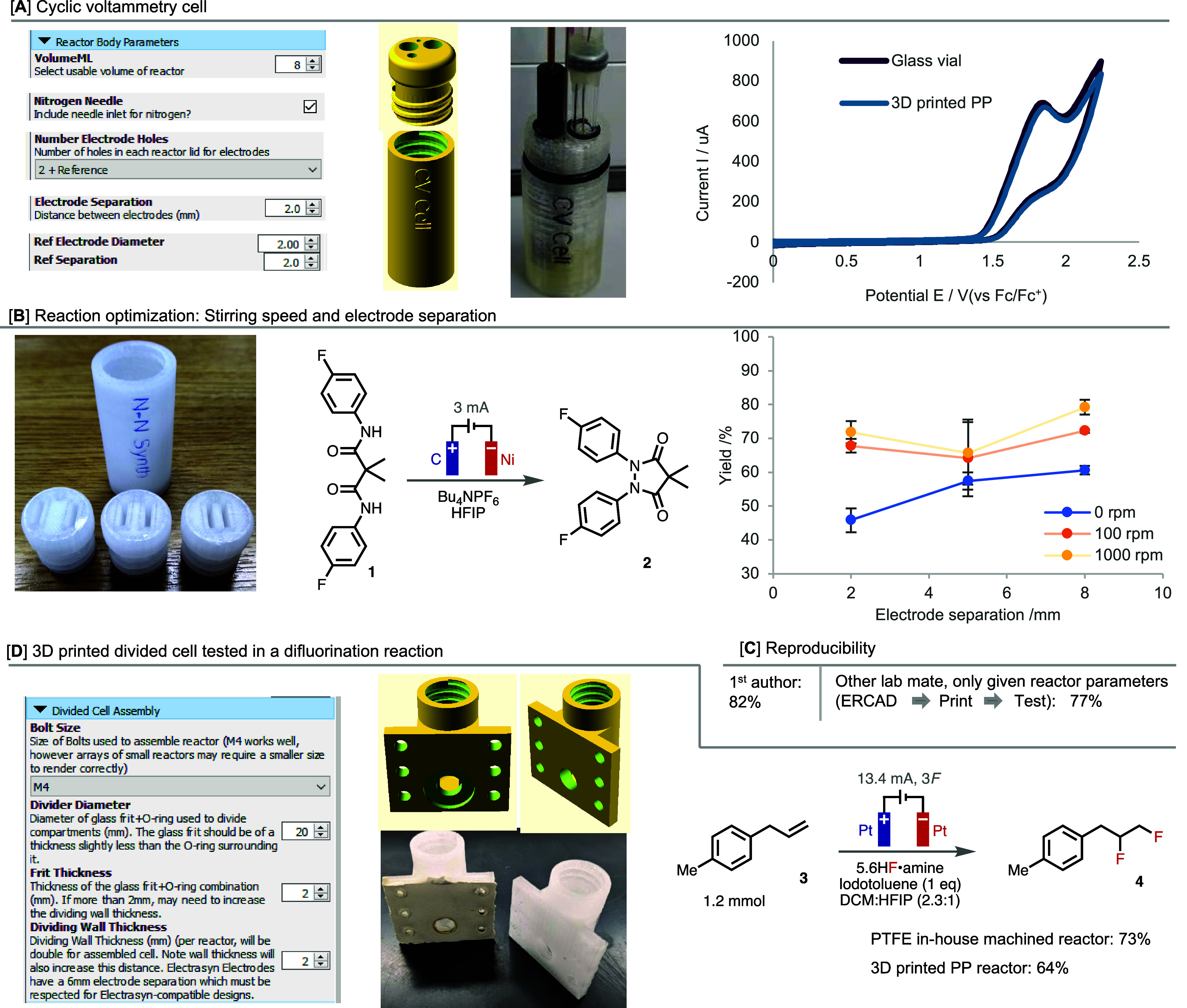
(A) ERCAD design parameters and printed reactor for CV experiments. (B) Investigation of reactor parameters with reactors designed through ERCAD (error bars shown in (B) are the standard errors on either side of the mean average of three runs). (C) Investigation of reproducibility on the reaction of **1** to **2** with ERCAD-designed reactors. (D) ERCAD design parameters, printed reactor, and alkene difluorination reaction performed in a divided cell. “Amine” refers to a mixture of NEt_3_ and pyridine, which is derived from mixing commercially available 3HF·NEt_3_ with py·9HF.

ERCAD can design a variety of electrochemical reactor-types and configurations (vide infra), without the user requiring any knowledge of CAD or the underlying codebase. Each reactor is a two-part construction, consisting of a body and lid (Figure 2B), through which the electrodes and other connections to the reactor are made. The lid can be configured to be a simple screw thread lid or be push-fit (Figure 2Bii) and will automatically resize to suit the reactor diameter (Figure 2Biii). The number, relative position, and shape of electrodes can vary (Figure 2Biv), which allows parameters, such as the interelectrode distance and the electrode size, to be easily adjusted with only a small percentage of the overall reactor needing to be reprinted when changes are made. Rather than the electrodes being inserted through the lid, the wires can be inserted through a septum on the lid (Figure 3Bv). Inert atmosphere can be provided with an N_2_ line, and spring-loaded pins can be pierced through the lid to create electrical contact points (see the Supporting Information (SI) for full details and Figure 2Bvi).^57^

### Benchmarking Reactors

In principle, the ERCAD-generated reactor CAD files can be used for any 3D printing method, as well as other computer-controlled machining techniques, and can be produced in-house or sent to external commercial fabricators. The reactors can also be constructed from any material, including engineering-grade materials, such as PEEK or stainless steel. However, we have focused on 3D printing of polypropylene reactors using a commercially available “desktop” FDM (fused deposition modeling) 3D printer. This technique allows reactors to be designed and manufactured within the same day, without knowledge of traditional subtractive manufacturing techniques, and at low cost.^24^ With compatibility to most solvents relevant to electrosynthesis (DCM and THF are documented to swell at elevated temperatures),^58,59^ polypropylene provides a good balance between chemical resistance and printability.^56^

To demonstrate the functionality of ERCAD, a variety of electrochemical reactor-types were designed, printed, and tested. The print settings that were used are given in the SI, which provided reactors that were found to be leak-free after filling with reaction solvent for 16 h. Nonetheless, secondary containment is always recommended when synthetic reactions are performed in 3D-printed reactionware.

A cell for cyclic voltammetry (CV) studies was first explored. Within ERCAD, a CV cell appropriate for a small volume experiment (8 mL) was created, and the dimensions of the electrodes used were specified. An additional port on the lid was also included, through which tubing could connect the cell to a Schlenk line to maintain an N_2_ atmosphere. A CV experiment under standard conditions demonstrated a similar performance between the 3D-printed cell and a commercially available glass/PTFE CV cell, as shown in Figure 3A.

ERCAD was then used to design a simple undivided cell. We opted to test a reported N–N dimerization reaction, which the publishing authors found to be sensitive to a variety of parameters such as current density and electrolyte concentration. Therefore, we considered this reaction to be a strong candidate for reactor-based optimization.^60^ We printed a series of lids in order to study the sensitivity of the reaction to the stirring speed and electrode separation distance. This exercise demonstrated the yield was strongly influenced by both of these parameters (Figure 3B). Through this, we found a reactor-type and conditions that were superior to those published, which were found to exhibit excellent reproducibility over 8 runs (see the SI).

To demonstrate broader reproducibility, a group member unrelated to the project was tasked with reproducing the experiment after being given only the reactor parameters and reaction conditions supplied in the SI of this paper. They successfully utilized ERCAD to design the reactor, print it, and run the reaction (Figure 3C). The yield observed was comparable to that originally observed. Beyond the simple action of sharing the print files themselves, this also demonstrates the applicability of ERCAD to produce reactors for sharing.

We then applied ERCAD to design a divided cell, which after printing in polypropylene proved leak-proof upon assembly. We wanted to test this reactor under demanding conditions, so we chose the oxidation of tolyl iodide to tolyl difluoro-λ-iodane in a solution of DCM, HFIP, NEt_3_·3HF, and py·9HF. As polypropylene is documented to swell with DCM (at least at elevated temperatures)^58,59^ and considering the corrosive nature of HF solutions, we considered these reaction conditions to be an especially strong test for the reactor. For practicality purposes, we monitored its subsequent reaction with an alkene to give the stable vicinal difluorinated alkane.^24,61^ Pleasingly, similar yields were observed between the 3D-printed divided cell and one that had been machined from a block of PTFE using traditional subtractive manufacturing techniques (Figure 3D). We did not observe any incompatibility (swelling, leaching, or corrosion) with the polypropylene reactor under these conditions, which may be due to the temperature of use or the fact that neat DCM was not used. The ability to rapidly prototype the 3D-printed reactors mean further improvements could be made if required. Nevertheless, this experiment demonstrates the ability to rapidly and inexpensively prototype a reactor to test its parameters before committing to machining a more expensive, but potentially more robust, PTFE cell.

**Figure 4:**
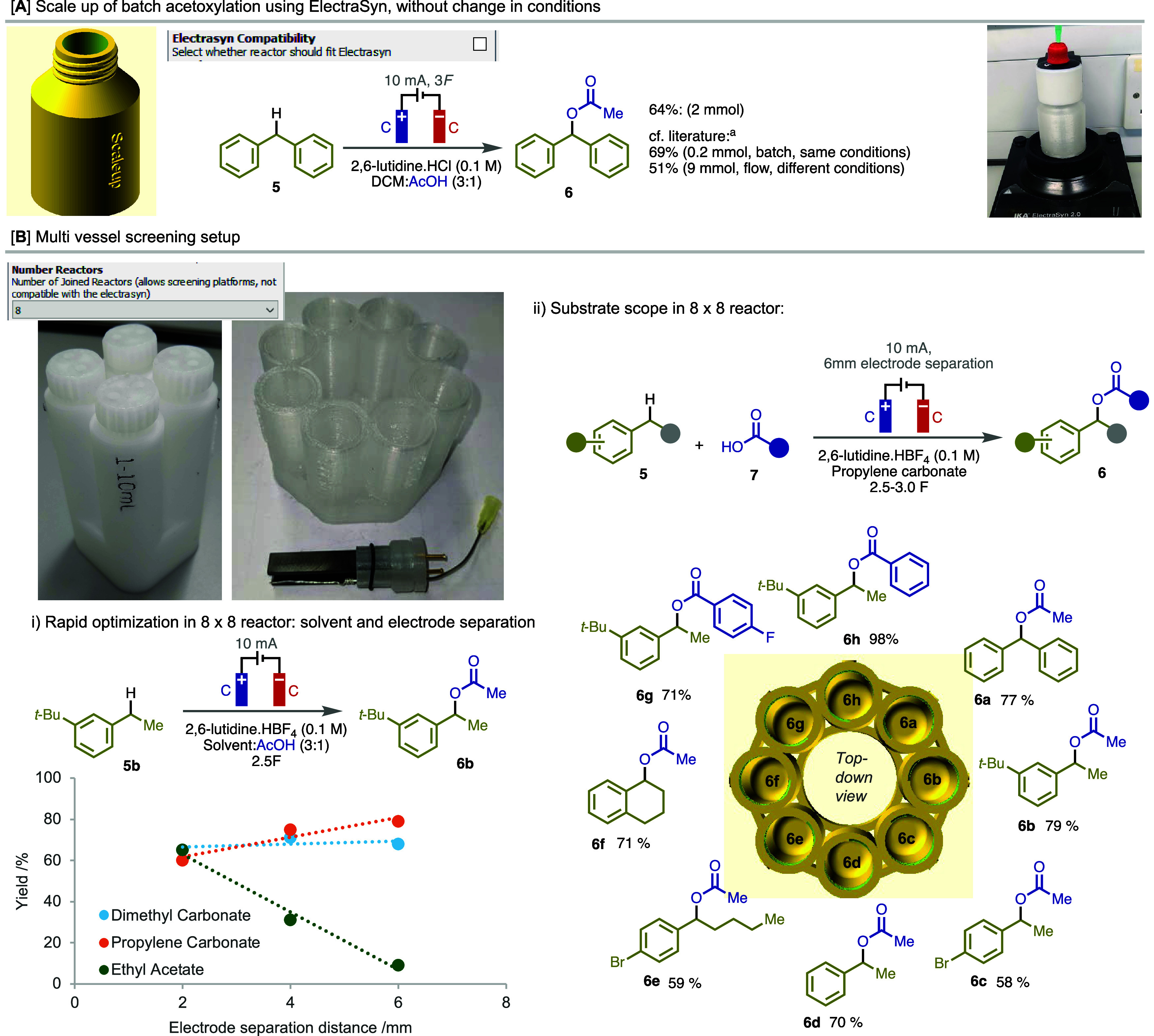
(A) Scaleup of a batch benzylic acetoxylation reaction using ERCAD-designed reactors. (B) Multiple reactor monoliths designed for (i) screening reaction conditions and (ii) investigating substrate scope. Legend: (a) see ref (62).

ERCAD includes the option to produce custom reactors that are compatible with the IKA Electrasyn platform. This option extends the functionality of this instrument: for example, to reactors that are of larger volume than are commercially available. For this, we tested a benzylic acyloxylation reaction, which was originally reported on a 0.2 mmol scale using a commercially available 5 mL reactor. A 10× scale-up was achieved using flow chemistry, but a reoptimization of the reaction conditions was required and a decreased yield was observed.^62^ Using ERCAD, we were able to rapidly design a 40 mL reactor, which was printed and used to enable the acyloxylation to be run on a 10× scale (2 mmol) at the same concentration. Again, no compatibility issues were detected with the use of DCM under these conditions. This setup gave a yield comparable to that published (Figure 4A), without the need to reoptimize the electrolyte, current density, or charge passed, as was previously necessitated when scaling up using flow chemistry.

The ability to study reactor parameters and reaction conditions in parallel should be useful to understand their sensitivity as well as optimize conditions. Running these optimization experiments rapidly and in parallel is not easily performed using conventional setups. However, using ERCAD, up to 8 reactors can be put together in a setup design, and so a screening setup consisting of 8 × 8 mL reactors was easily designed and then printed. To demonstrate its utility, we opted to undertake a reoptimization of the solvent and electrode separation distance in a benzylic C(sp^3^)–H acetoxylation,^62^ with the aim of employing a greener solvent than the DCM that was originally used,^63,64^ while simultaneously improving reaction performance (Figure 4Bi). Ethyl acetate, dimethyl carbonate, and propylene carbonate were identified as potentially suitable greener solvents to test. Interestingly, while smaller electrode distances were favored with ethyl acetate, propylene carbonate performed better with larger distances and dimethyl carbonate was relatively insensitive to this parameter. The yield was far more sensitive to electrode separation distance in ethyl acetate than in the others, which is likely due to a poorer conductivity of the electrolyte solution.

Following this process of reoptimization, several additional substrates were tested under the new conditions using the same setup. The yields obtained for all substrates proved comparable between those run in the 3D printed screening setup using a greener solvent and those originally reported using the ElectraSyn.^62^

## Summary and Conclusions

In conclusion, we have developed the Electrochemical Reactor Computer Aided Design (ERCAD) tool that provides a simple way to easily produce reactor designs for 3D printing. ERCAD removes the need for chemists to become fluent in Computer-Aided Design in order to create custom reactors, replacing it with intuitive menus, where reactors are produced in chemistry-relevant terms. The application of ERCAD has been demonstrated for the generation of CV cells, undivided and divided cells, including compatibility with the Electrasyn potentiostat and electrodes. ERCAD has also been applied to a multireactor screening setup, in which a greener and higher yielding set of conditions were optimized and then used for a small scope of substrates. The workflow that ERCAD now offers enables the rapid investigation of reactor parameter sensitivity in a particular reaction, reactor optimization, prototyping, and iteration, and high reaction reproducibility. The creation of ERCAD should strengthen the case for 3D-printed reactors to be an effective technique to manufacture bespoke electrochemical reactors.

## Data Availability

ERCAD files are available at the University of Bristol data repository, data.bris, at 10.5523/bris.2ko5vmzjsp1z1290n89glwgz3r.

## Supporting Information Available

Software, hardware, materials for 3D printing and reactor assembly, OpenSCAD installation and use of the ERCAD package, code appendix, cyclic voltammetry procedures, synthetic procedures, and NMR spectra (PDF)
