# Supplementary material for: ERCAD: A Parametric Reactor Design Tool That Enables Rapid Prototyping and Optimization of Electrochemical Reactors through 3D Printing
Source: ACS Cent Sci. 2024 Sep 27;10(11):2028–35. doi: 10.1021/acscentsci.4c00988 (PMC11613433; doi:10.1021/acscentsci.4c00988)
Supplement: Supplementary file 1 — oc4c00988_si_001.pdf [file oc4c00988_si_001.pdf]

ERCAD: A Parametric Reactor Design Tool that Enables Rapid Prototyping and Optimisation of Electrochemical Reactors through 3D Printing

David M. Heard[1], Sam W. Deeks[1], Alastair J.J. Lennox[1]*

Email: a.lennox@bristol.ac.uk

Author Affiliations: 1. School of Chemistry, University of Bristol, Cantock's Close, Bristol, UK, BS8 1TS

Supplementary Information

General Experimental Details. *3*

Software, Hardware & Materials for 3D Printing & Cell Assembly 4

3D Printing of Electrochemical Reactors *5*

Installation of OpenSCAD and use of the ERCAD package *8*

Synthetic Procedures *10*

Cyclic Voltammetry Studies 10

Electrode Separation Studies 13

Anodic N-N Dimerisation Parameter Study 15

Divided Cell Assembly 21

Difluorination of Alkenes 23

Electrasyn Scale-up Studies 25

Scaleup of Acetoxylation Reaction 27

Screening Unit – Acetoxylation/Acyloxylation Reaction 30

Screening Setup - Airtight Lid Assembly 32

General Procedure for Acetoxylation Reaction 34

Acyloxylation of 1-*tert*-butyl-3-ethylbenzene 41

References *68*

Code Appendix *44*

ERCAD 44

Reactor Presets 64

General Experimental Details.

All reagents were sourced from commercial suppliers and were used without further purification. Unless otherwise indicated, all reactions were performed open to air, with reagent-grade solvents and no attempt to exclude water. When necessary, dichloromethane, tetrahydrofuran, diethyl ether and toluene were dried using an Anhydrous Engineering alumina column drying system situated in the University of Bristol School of Chemistry. Triethylamine tris (hydrogen fluoride) and 1,1,1,3,3,3-hexafluoro-2-propanol (HFIP) were purchased from Fluorochem and used as received. Deuterated solvents for NMR analysis were purchased from Sigma Aldrich. Flash column chromatography was performed according to the procedures used by Still *et al.*[1] using silica gel 60 (Fisher Scientific or Aldrich) and a suitable eluent. TLC analysis was performed with aluminium backed silica TLC plates (Merck-Kieselgel 60 F_{254}) with a suitable solvent system and was visualised using UV fluorescence (254 & 366 nm) and/or developed with potassium permanganate solution.

Infra-red spectra were recorded on a PerkinElmer Spectrum 100 FTIR with an ATR accessory and frequencies are reported in wavenumbers (cm^{-1}). ^{1}H and ^{13}C NMR spectra were recorded using either a Jeol ECS 400 MHz or Varian 400-MR (400 MHz) spectrometer at ambient temperature. Chemical shifts (δ) are quoted in parts per million (ppm) and coupling constants (J) are in Hertz (Hz), rounded to 0.5 Hz intervals. Where NMR yields are used, a delay time of 40 seconds (^{1}H) or 20 seconds (^{19}F) was applied. Two-dimensional NMR techniques (HSQC, COSY, HMBC) were used routinely for structural assignment. Residual solvent peaks were used as the internal reference for proton and carbon chemical shifts. High-Resolution Mass Spectrometry was performed on a Bruker microTOF II using Electrospray ionisation. Samples were submitted in MeOH, or $CHCl_3$. Specific rotations ($[\alpha]_T^D$) were measured on a Bellingham and Stanley Ltd. ADP220 polarimeter and are quoted in (° ml) (g dm)$^{-1}$.

Electrochemical reactions were performed with either an IKA Electrasyn 2.0 or a Palmsens MultiPalmSens4. Electrasyn-compatible electrodes were used for all electrochemical reactions, and were purchased from IKA. Electrodes were wiped clean with a Kimwipe™, sonicated for 0.5 h in a 1:1 acetone:ethanol solution, and then dried overnight at 80 ℃.

Warning: Use of HF reagents

The hazards of hydrogen fluoride solutions are well known (See SDS at https://www.sigmaaldrich.com/catalog/product/aldrich/184225) and should only be handled by experienced personnel. Personal protection is of the utmost importance. It is advised to wear two pairs of nitrile gloves when handling, and if the gloves encounter HF, they are removed **immediately,** the area affected washed thoroughly with Hexafluorine solution™ (http://www.medicalcare.se/diphoterine-and-hexafluorine), calcium glucanoate gel is applied to the area and medical attention is sought. It is advised that Hexafluorine Solution™ and calcium glucanoate gel are kept nearby for personal use.

Software, Hardware & Materials for 3D Printing & Cell Assembly

All cells were designed with the ERCAD tool in OpenSCAD (Version 2021.01, available at: https://openscad.org/downloads.html), and exported as STL files. Optimisation of the design for 3D printing was not found to be necessary to produce a viable reactor but was achieved through the Remesh feature in AutoDesk Fusion360 where necessary to reduce printing times. The STL files were converted to GCode using Simplify3D and printed on a MakerGear M3-ID 3D Printer, using 1.75 mm transparent polypropylene filament supplied by RS-Pro (RS Stock No. 174-0056). Slicing and Printing settings are detailed below. Divided cells were assembled using M4 bolts obtained from a local hardware store, and O-rings were supplied by Duratool (Part No. D01887). The same O-rings were used to form a seal between reactor lid and reactor body sections. Nafion™ membranes were used for divided cells, and where necessary an additional sealing gasket was cut from EPDM sponge sheeting (RS Stock No. 103-4061). The design and construction of platinum electrodes used in this study has been previously reported.[2] PCB Surface Mount Spring-Loaded Pin (0937-0-15-20-77-14-11-0) are manufactured by Mill-Max and can be purchased from mouser.co.uk.

3D Printing of Electrochemical Reactors

In general, any FDM (Fused Deposition Modelling) 3D Printer which can extrude PLA or ABS (the most common plastics used in FDM printing) will be capable of reaching the temperatures required to extrude polypropylene. A MakerGear M3-ID 3D Printer was used for all reactors tested in this study, although a Creality Ender 3v2 Printer was also capable of producing prints of a similar quality. The ability to remove the print bed for replacement with a polypropylene sheet or covering with polypropylene tape is an important feature for ensuring good adhesion of the reactor to the print bed during the printing process. Step-by-step guides to the 3D printing of polypropylene have previously been published, and include troubleshooting advice.[3]

Reactors were printed with 1.75 mm diameter polypropylene filament. The Reactor body and lid STL files are produced by ERCAD with a ‘perfect’ fit, meaning the lids are oversized for assembly. This is solved by printing the reactor bodies at 100% scale, and the lids at 95% scale. Depending on the size of the reactor and quality of the printer used, this ratio may need to be fine-tuned to ensure a good fit. Parts were printed onto a glass print bed covered with polypropylene packing tape to aid adhesion. Key parameters for a successful print are given in table **S1**.

During testing of the reactors, we found that an excellent fit is required between the lid and reaction vessel to achieve an air-tight reactor, and this may only be possible with high-end 3D printers or by fine-tuning the scale at which reactor lids are printed. To improve the quality of seal between the printed reactor body and lid, two options were added to the ERCAD design tool. Firstly, replacing the screw-thread lids with a push-fit lid allows for improved fit between the components, reducing the amount of surface area contact needed to create a good seal, and allowing increased pressure against an O-ring to be applied to improve the seal. A thin disc of solid material can also be printed on the top of the reactor lid, sealing the holes for the electrodes from the outside of the reactor. The protocol for creating electrical connections through this seal is detailed *vide infra*.

Safety Note:

Solvents compatible with polypropylene are reported to include acetic acid, acetone, acetonitrile, dichloromethane (at room temperature, and may swell), ethanol, ethyl acetate, methanol, pyridine, tetrahydrofuran, and water. (It is recommended to consult a solvent compatibility chart such as https://www.ineos.com/globalassets/ineos-group/businesses/ineos-olefins-and-polymers-usa/products/technical-information--patents/ineos-pp-chemical-resistance-guide.pdf before use) Polypropylene experiences some attack from chloroform, diethyl ether and hexane, and is incompatible with aromatic solvents. The compatibility of polypropylene with HFIP and propylene carbonate, the other solvents used in our study, has not been extensively studied, however within our work no significant deterioration of the reactors were observed.

3D Printed objects cannot be assumed to be leak-free without testing. Whilst we experienced high rates of successful prints, an imperfect printer setup (for example a misaligned, or partially blocked nozzle) can significantly reduce the quality of printed reactors and lead to leaks.

After each reactor was printed and visually inspected for flaws, it was filled with reaction solvent and monitored for 16 h to ensure no leaks or degradation by reaction solvent had occurred. Nevertheless, secondary containment and inspection of the reactor before re-use is essential when performing synthetic reactions in 3D printed reactionware.

Regarding impurities and leaching: the spectroscopic analysis of crude reaction mixtures gave no indication of consistent impurities across different reactions, which would indicate leaching from the reactors. Although leaching of additives cannot be ruled out, their presence or effect were not detected.

Parameter	Value	Parameter	Value
Nozzle Temperature	240 °C	Primary Layer Height	0.2 mm
Bed Temperature	80 °C	Top Solid Layers	5
Nozzle Diameter	0.35 mm	Bottom Solid Layers	5
Extrusion Multiplier	1.05	Outline/Perimeter Shells	4
Retraction Distance	0.50 mm	First Layer Height	70%
Extra Restart Distance	0.03 mm	First Layer Speed	50%
Infill Percentage	100%	Skirt Layers	2
Fill Pattern	Rectilinear	Skirt Offset	0 mm
Outline Overlap	15%	Skirt Outlines	15
Infill Extrusion Width	100%	Default Printing Speed	3600.0 mm/min
Minimum Infill Length	5.0 mm	Default Printing Speed*	2000 mm/min
Infill Angle Offset	±45°	Outline Underspeed	60%
Support	None	Solid Infill Underspeed	80%
Fan Speed	60%	X/Y Axis Movement Speed	5000 mm/min
Fan Speed*	0%	Z Axis Movement Speed	1200 mm/min
Seam Position	Random		

Table S1. Print Settings used for 3D Printing of Reactors and Lids.* Altered settings for printing screening setups

Installation of OpenSCAD and use of the ERCAD package

1. OpenSCAD is freely available from https://openscad.org/downloads.html and is installed according to the instructions on the website.
2. ERCAD can be downloaded from data.bris, at https://doi.org/10.5523/bris.2ko5vmzjsp1z1290n89glwgz3r.
   DOI: 10.5523/bris.2ko5vmzjsp1z1290n89glwgz3r. It consists of two files, ERCAD.scad and ERCAD.json. Download both files and place in the same folder.
3. Open ERCAD.scad in OpenSCAD. The window should open as shown below.

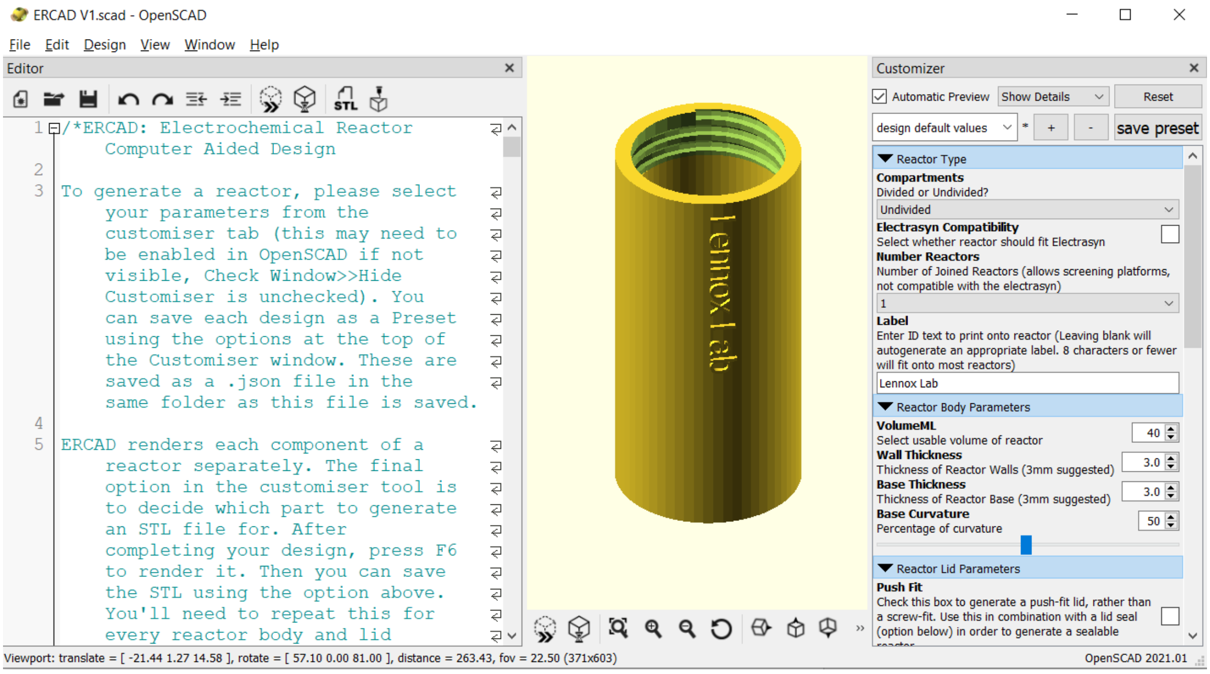

Figure S1. First screen encountered when opening ERCAD

4. The editor window (Figure **S1**, left) can be ignored, or the X in the top right corner clicked to remove it from the screen. If the Customizer window (Figure **S1**, right) does not show initially, it can be enabled from the Window menu. The Console and Error log can similarly be removed if desired.
5. Unless you are running high-end computer hardware, untick the “Automatic Preview” option at the top of the Customizer window.
6. To design a reactor, expand each tab within the Customizer window, and enter your desired settings. Few reactor designs will require you to enter a value in all areas, guidance is provided within ERCAD. Not all combinations of values will lead to a valid reactor design, for example it is not possible to generate a divided cell compatible with the Electrasyn.[4]

7. At the bottom of the Customizer window is an option to choose which part to render (An Undivided Cell, two different halves for a divided cells, and a lid). Select the desired component, and select the Part Quality from the dropdown menu. This setting will determine whether part quality or rendering speed is prioritised.
8. Press F6 to render the chosen reactor (or the render button on the menu). This may take several minutes. The highest quality parts may take several minutes to render within OpenSCAD, so it is suggested to use a lower quality setting whilst designing a reactor, and only render the final design at the higher quality setting.
9. Press F7 to export the rendered design as an STL file (or the STL button on the menu). Save the generated part, and then return to ERCAD to render and export any other components required.
10. The STL files can now be used for 3D printing.
11. Designed reactors can be saved from the Customizer window by entering a name into the topmost text box, and selecting Save Preset. The reactor design will be added to the ERCAD.json file and can be selected from the dropdown menu.

Note: If sharing reactors designed in ERCAD, the .json file containing the reactor design must have the same filename as the .scad file containing ERCAD, otherwise the settings file will not be recognised.

Synthetic Procedures

Cyclic Voltammetry Studies

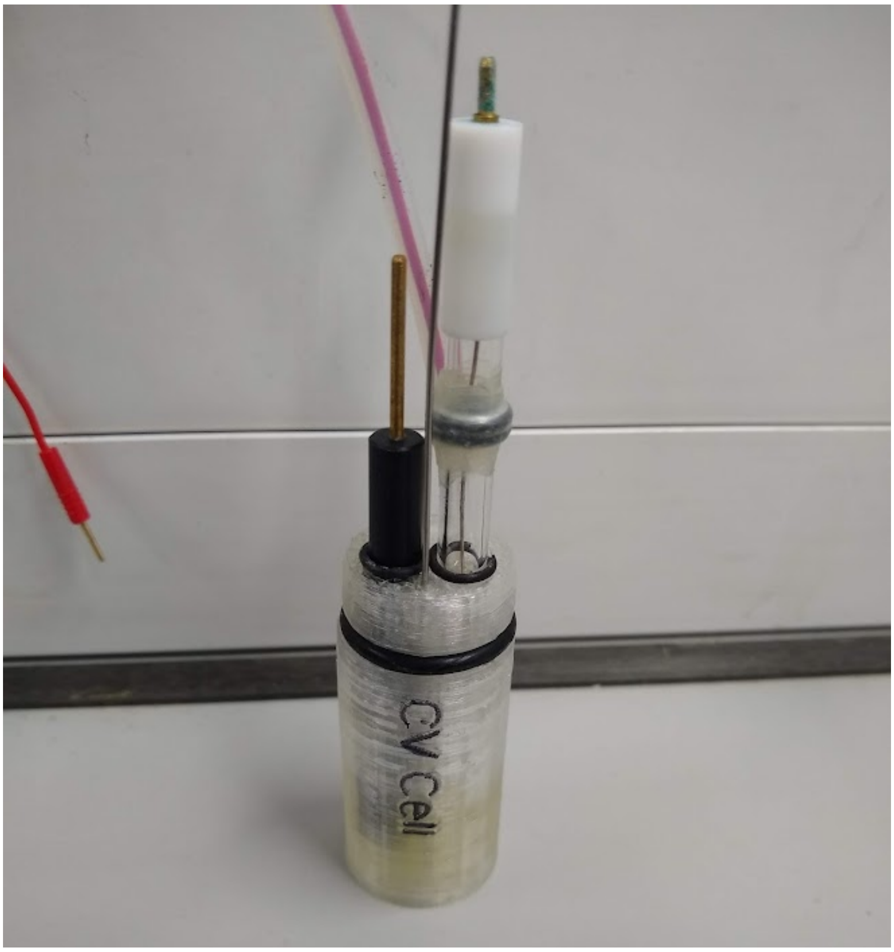

Figure S2. 3D Printed Cyclic Voltammetry cell used for the collection of the CV data presented in Figure **S3**. A Ferrocene solution is visible through the walls of the cell.

Parameter	Value	Parameter	Value
**Reactor Type**		**Reference Electrode**	
Divided or Undivided?	Undivided	Ref Electrode	N/A
Electrasyn Compatible?	No	Ref Electrode Diameter	3.5
Number of Reactors	1	Ref Separation	1.5
Label	"CV Cell"		
**Reactor Body Parameters**		**Divided Cell Assembly**	
VolumeML	8	Bolt Size	N/A
Wall Thickness	2	Divider Diameter	N/A
Base Thickness	2	Frit Thickness	N/A
Base Curvature	25	Dividing Wall Thickness	N/A
**Reactor Lid Parameters**		**Other Parameters**	
Push Fit	No	HR Ratio	4
Lid Seal	No	Seal Thickness	N/A
Lid Config	Electrodes Only	Parts to Render	Lid & Reactor
Nitrogen Needle	Yes	Part Quality	High
Suba Size	N/A		
Number Electrode Holes	2+Reference		
Electrode Hole Shape	Circular		
Electrode Hole Diameter	6.5		
Rectangular Electrode X	N/A		
Rectangular Electrode Y	N/A		
Electrode Separation	1.5		

Table S2. ERCAD parameter settings for the CV cell pictured in **Figure S2**.

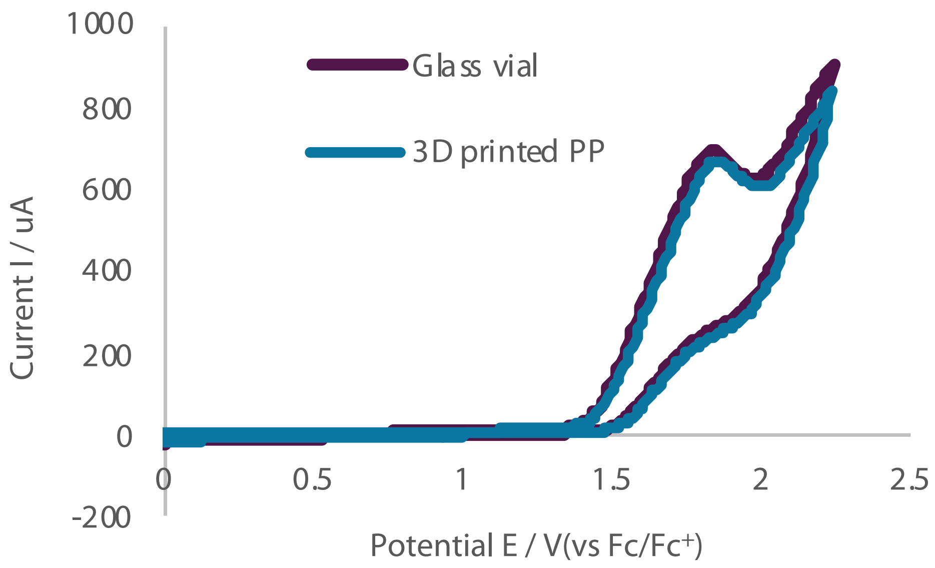

Figure S3. Cyclic Voltammogram of 1-tert-Butyl-4-ethylbenzene and the background signal in both a glass vial and a polypropylene printed vial. Substrate (10 mM) in Acetonitrile (anhydrous, degassed with a stream of nitrogen) with $TBAPF_6$ electrolyte (0.1 M). Glassy carbon disc working electrode, platinum wire counter electrode, Ag/$AgNO_3$ reference electrode. 0.1 Vs^{-1} scan rate. Referenced vs Fc/Fc^+.

Data obtained in good accordance with that previously obtained with a glass CV cell fitted with a commercial PTFE lid.[5]

Electrode Separation Studies

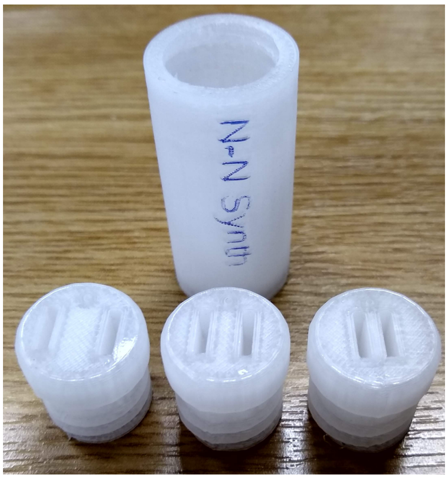

Figure S4. 3D printed cell used for the optimising electrode separation. Data presented in **Figure S5**.

Parameter	Value	Parameter	Value
**Reactor Type**		**Reference Electrode**	
Divided or Undivided?	Undivided	Ref Electrode	N/A
Electrasyn Compatible?	No	Ref Electrode Diameter	N/A
Number of Reactors	1	Ref Separation	N/A
Label	"Undivided Reactor"		
**Reactor Body Parameters**		**Divided Cell Assembly**	
VolumeML	8	Bolt Size	N/A
Wall Thickness	2	Divider Diameter	N/A
Base Thickness	2	Frit Thickness	N/A
Base Curvature	25	Dividing Wall Thickness	N/A
**Reactor Lid Parameters**		**Other Parameters**	
Push Fit	No	HR Ratio	4
Lid Seal	No	Seal Thickness	N/A
Lid Config	Electrodes Only	Parts to Render	Lid & Reactor
Nitrogen Needle	No	Part Quality	High
Suba Size	N/A		
Number Electrode Holes	2		
Electrode Hole Shape	Electrasyn		
Electrode Hole Diameter	N/A		
Rectangular Electrode X	N/A		
Rectangular Electrode Y	N/A		
Electrode Separation	Variable		

Table S3. ERCAD parameter settings for the cell used to test electrode separation pictured in **Figure S4**.

Anodic N-N Dimerisation Parameter Study

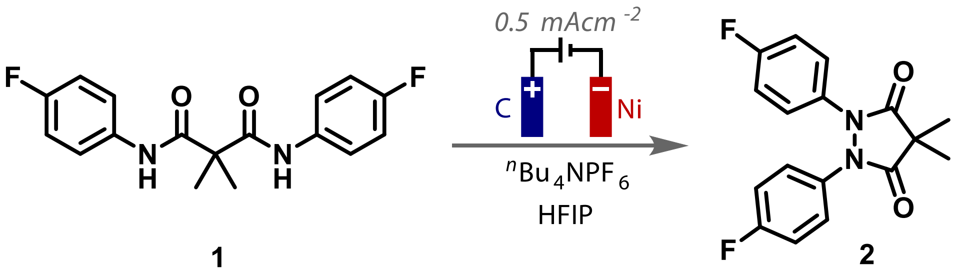

Adapted from a literature procedure.[6] To a 3D printed, 8 mL undivided cell equipped with a graphite anode and nickel cathode was added *N*,*N*-bis(4-fluorophenyl)-2,2-dimethylmalonamide (64.0 mg, 0.2 mmol, 1 equiv., prepared according to literature procedure)[6], tetrabutylammonium hexafluorophosphate (19.5 mg, 0.25 equiv.) and HFIP (3 mL). The reaction mixture was stirred at room temperature and then electrolysed (3 mA, 2.2 F). After electrolysis, the electrodes were rinsed with additional HFIP and then the reaction mixture was concentrated *in vacuo*, and a known quantity of NMR standard (1,3,5-trimethoxybenzene) was added. The reaction mixture was dissolved in d_4-MeOH and the amount of product formed was determined by ^{1}H NMR from the ratio of the product (7.41-7.36 (2H, m)) to the standard (6.08 (3H, s)). For characterisation purposes, the product obtained was purified by flash chromatography (eluting with 20:80% ethyl acetate:Pentane).

The following data are consistent with those previously reported.[6]

1,2-Bis(4-fluorophenyl)-4,4-dimethylpyrazolidin-3,5-dione

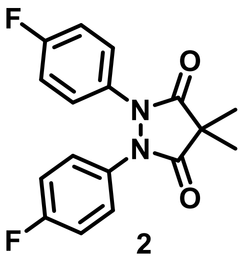

2

^{1}H NMR (400 MHz, $CDCl_3$) δ 7.29-7.24 (4H, m, Ar-H), 7.03-6.97 (4H, m, Ar-H), 1.48 (6H, s, CH_3)

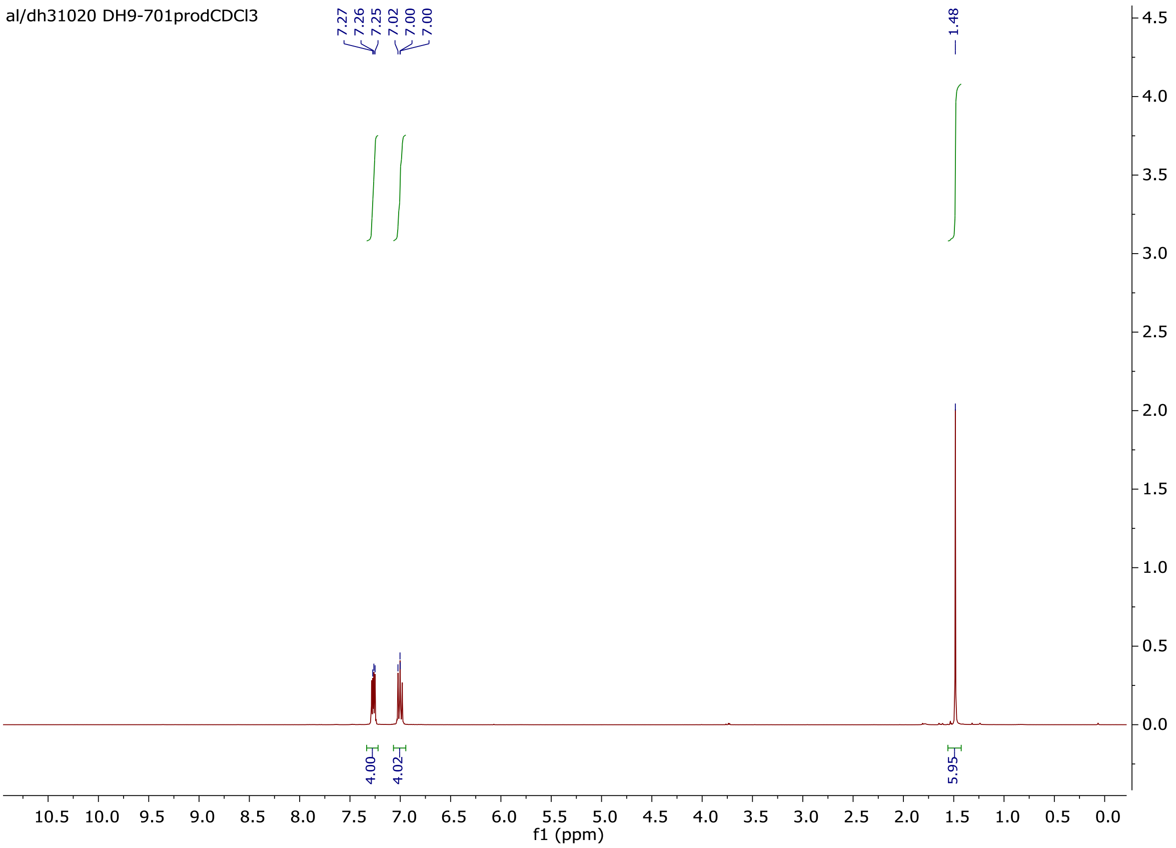

^{13}C NMR (101 MHz, CDCl$_3$) δ 174.4 (C=O), 161.0 (d, *J* = 247.0 Hz, Ar), 131.7 (d, *J* = 3.0 Hz, Ar), 124.7 (d, *J* = 8.5 Hz, Ar), 116.2 (d, *J* = 23.5 Hz, Ar), 44.3 (C), 21.8 (CH$_3$)

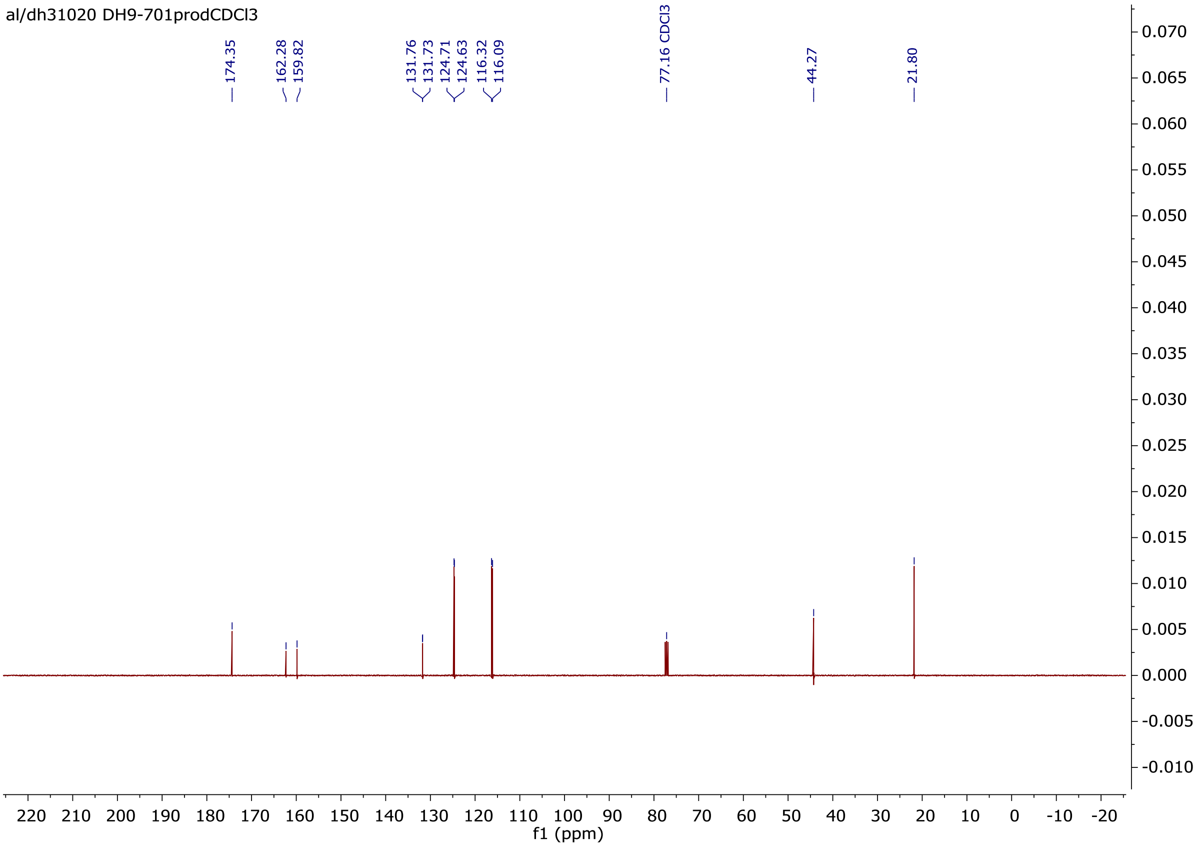

^{19}F (376 MHz, $CDCl_3$) δ -113.78

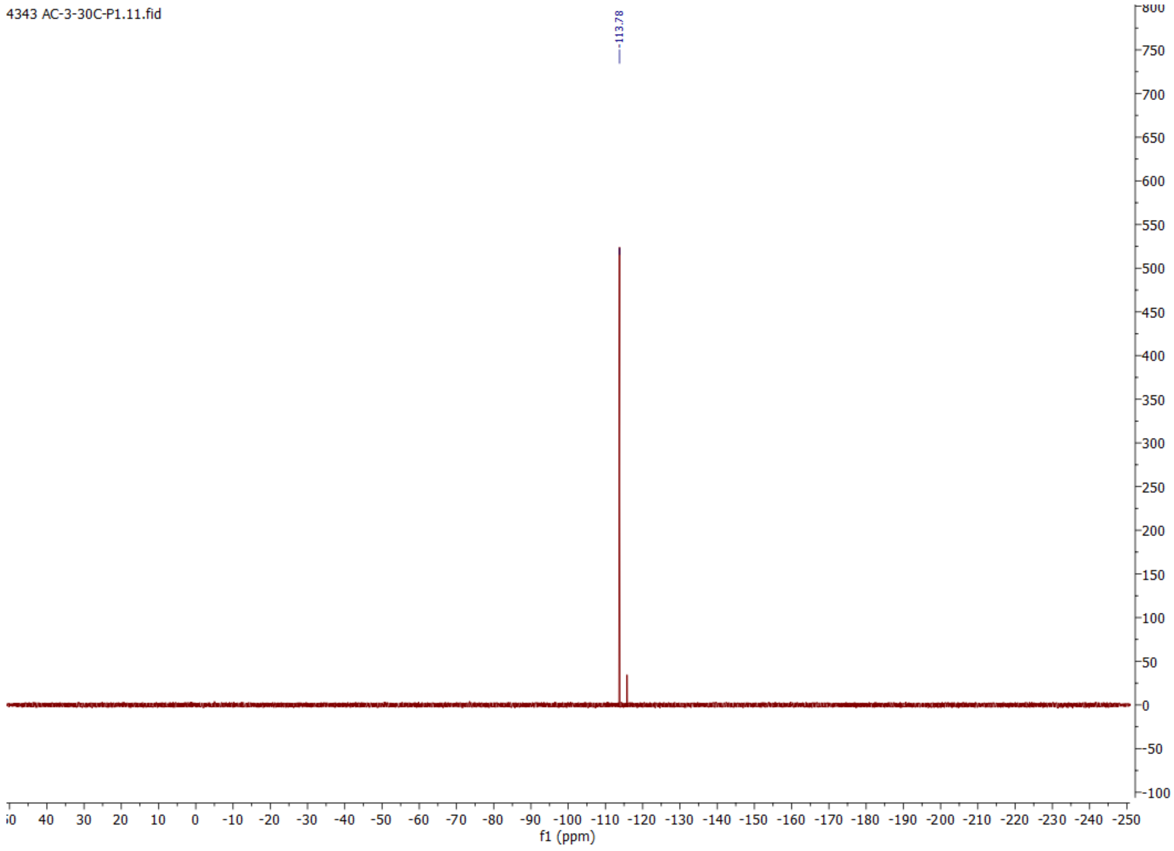

Repeatability of the reaction was established by running the reaction with a 2 mm electrode separation, 500 rpm stir rate. Yields are given in **Table S4**. Reactor parameters were then varied, and reactions run in triplicate. Yields are given in **Table S5**.

Table S4. Yields obtained in repeatability studies conducted on the anodic N-N dimerization reaction.

Yield 1 / %	Yield 2 / %	Yield 3 / %	Yield 4 / %	Yield 5 / %	Yield 6 / %	Yield 7 / %	Mean Yield / %
83.9	84.4	78.1	89.9	76.8	71.1	87.9	**81.7**

Table S5. Yields obtained when varying reactor parameters.

Entry	Stir Rate / rpm	Electrode Separation / mm	Yield 1 / %	Yield 2 / %	Yield 3 / %	Mean Yield / %
**1**	0	2	39.4	46.3	51.6	**45.8**
**2**	0	5	53.3	56.9	62.1	**57.4**
**3**	0	8	58.5	60.6	62.7	**60.6**
**4**	100	2	67.7	64.3	71.3	**67.8**
**5**	100	5	42.5	69.3	80.8	**64.2**
**6**	100	8	71.5	71.8	73.2	**72.2**
**7**	500	5	84.7	77.2	72.4	**78.1**
**8**	500	8	34.2	36.8	39.7	**36.9**
**9**	1000	2	67.9	78.4	69.0	**71.8**
**10**	1000	5	66.9	80.9	48.9	**65.6**
**11**	1000	8	78.5	75.8	83.2	**79.2**

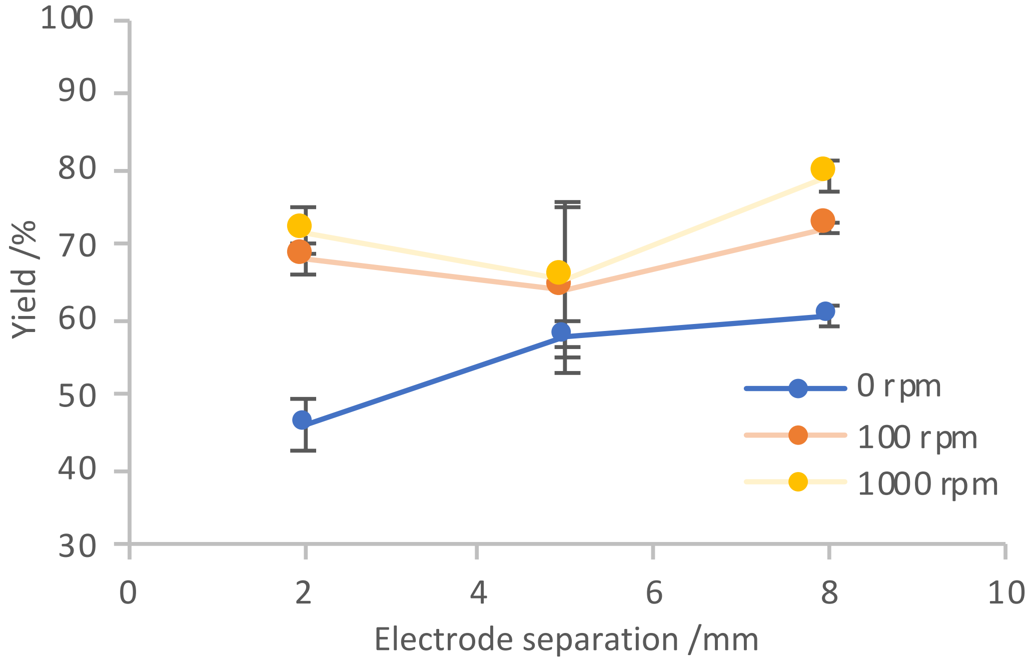

Figure S5. Yields for the N-N bond forming reaction with varying electrode separation and stirring rate. Error bars show standard error either side the mean average of three runs.

Divided Cell Assembly

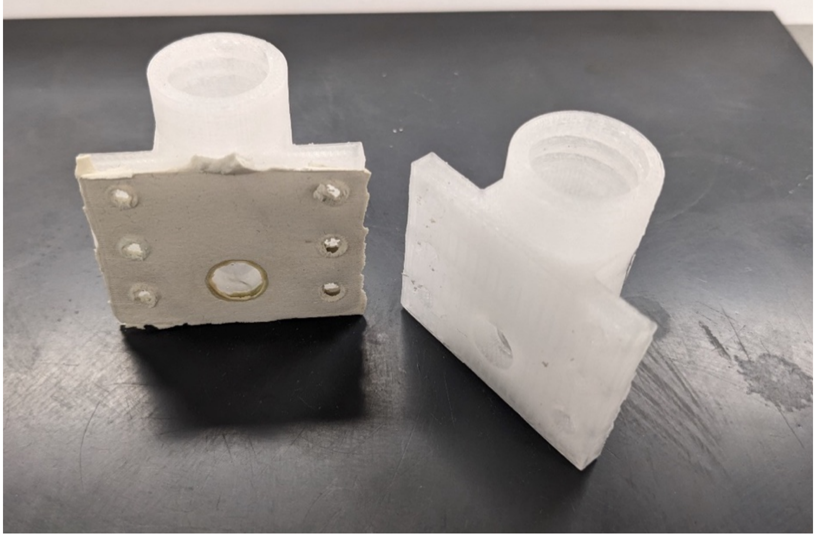

Figure S6: 3D-printed divided cell. Two compartments were fastened together using M4 nuts and bolts and separated by a Nafion™ membrane sandwiched between two layers of EPDM sponge sheeting (RS Stock No. 103-4061) foam cut to size.

Parameter	Value	Parameter	Value
**Reactor Type**		**Reference Electrode**	
Divided or Undivided?	Divided	Ref Electrode	N/A
Electrasyn Compatible?	No	Ref Electrode Diameter	N/A
Number of Reactors	1	Ref Separation	N/A
Label	"Divided Cell"		
**Reactor Body Parameters**		**Divided Cell Assembly**	
VolumeML	8	Bolt Size	M4
Wall Thickness	3	Divider Diameter	20
Base Thickness	3	Frit Thickness	0
Base Curvature	50	Dividing Wall Thickness	2
**Reactor Lid Parameters**		**Other Parameters**	
Push Fit	No	HR Ratio	4
Lid Seal	No	Seal Thickness	N/A
Lid Config	Electrodes Only	Parts to Render	Lid & Reactor
Nitrogen Needle	No	Part Quality	High
Suba Size	N/A		
Number Electrode Holes	1		
Electrode Hole Shape	Circular		
Electrode Hole Diameter	3		
Rectangular Electrode X	N/A		
Rectangular Electrode Y	N/A		
Electrode Separation	1.5		

Table S6. ERCAD parameter settings for the divided cell pictured in Figure **S6**.

Difluorination of Alkenes

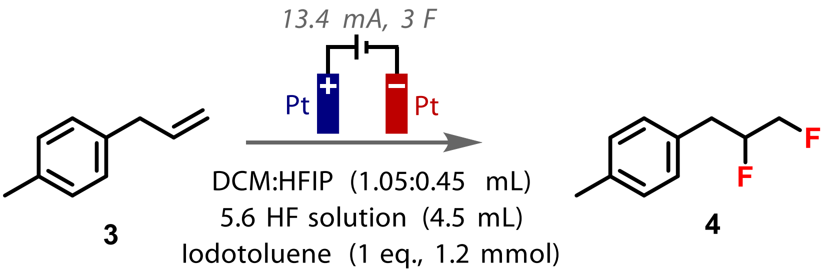

To each compartment of the 3D printed divided cell equipped with a Nafion™ membrane sandwiched between two pieces of EPDM foam cut to size, stirring bars, CH_2Cl_2 (1.05 mL), HFIP (0.45 mL) and 5.6 HF stock solution (4.5 mL, prepared according to literature.[2]) were added. To the anodic compartment, 4-iodotoluene (1 eq., 1.2 mmol, 262 mg) was then added. Each compartment was then capped and wrapped in parafilm. A platinum electrode was inserted into each compartment, and the reaction was subjected to electrolysis (13.4 mA, 3 F/mol, 7.2 hrs). The electrodes were then removed, **3** (1 eq., 1.2 mmol) was then added to the anodic compartment and then caps were wrapped in parafilm to seal the reaction. After stirring overnight, the contents of each compartment were mixed and quenched with 100 mL of cold (0 ºC) saturated aqueous $CaCO_3$ solution This was stirred for 1 hour until the aqueous layer measured pH 7. The mixture was extracted into CH_2Cl_2, dried with Na_2SO_4 and evaporated *in vacuo*. A known quantity of NMR standard (4,4′-difluorobiphenyl) was added and the reaction mixture dissolved in d_6-DMSO.

^{1}H NMR yields were attained because assessments and comparisons of the reactors were the primary focus. The amount of product formed was determined by ^{1}H NMR from the ratio of the fluorine product peak (-232.00 (1F, m)) to the standard (-116.06 (2F, m)).[2]

1-(2,3-difluoropropyl)-4-methylbenzene

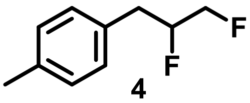

4

Title compound **4** was prepared from 4-allyltoluene following the divided cell difluorination experimental with a recorded NMR yield of 64%. The amount of product formed was determined by ^{19}F NMR from the ratio of the fluorine product peak (-232.00 (1F, tdd)) to 0.5 equivalents of 4,4′-Difluorobiphenyl (-116.06 (2F, m)).

The characteristic product peak is in agreement with the literature, -231.98 (1F, tdd, J = 46.9, 22.31, 14.2 Hz).[2]

^{19}F NMR (376 MHz, d_6-DMSO):

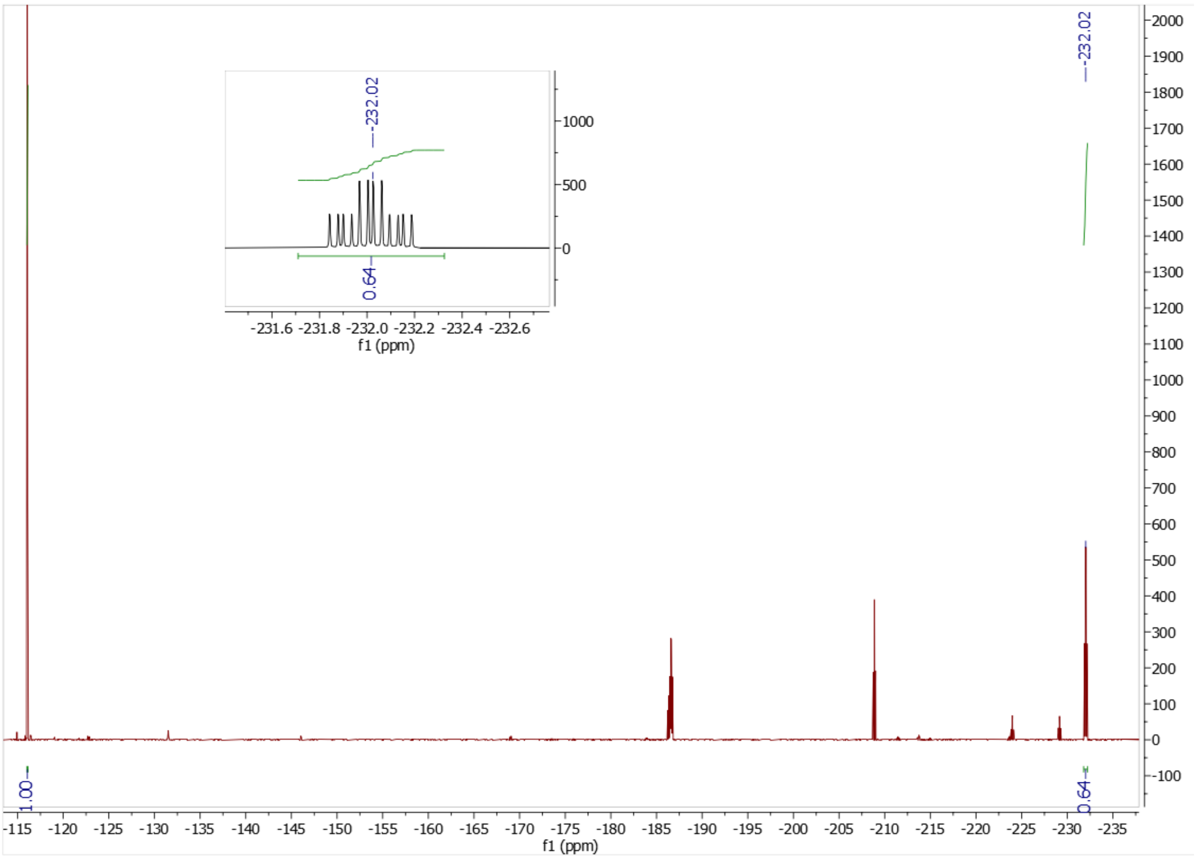

Electrasyn Scale-up Studies

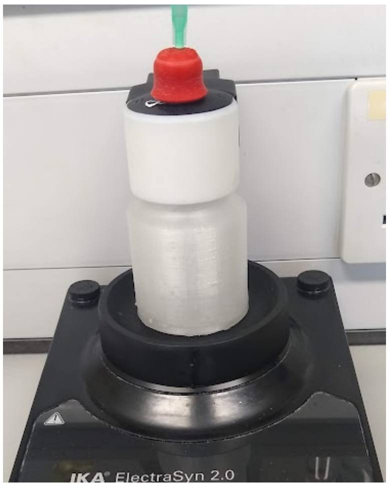

Figure S7. 3D Printed Electrasyn compatible cell used to scale the benzylic acetoxylation reaction.

Parameter	Value	Parameter	Value
**Reactor Type**		**Reference Electrode**	
Divided or Undivided?	Undivided	Ref Electrode	N/A
Electrasyn Compatible?	Yes	Ref Electrode Diameter	N/A
Number of Reactors	1	Ref Separation	N/A
Label	“Electrasyn Compatible Cell”		
**Reactor Body Parameters**		**Divided Cell Assembly**	
VolumeML	40	Bolt Size	N/A
Wall Thickness	2.0	Divider Diameter	N/A
Base Thickness	2.0	Frit Thickness	N/A
Base Curvature	20	Dividing Wall Thickness	N/A
**Reactor Lid Parameters**		**Other Parameters**	
Push Fit	N/A	HR Ratio	N/A
Lid Seal	N/A	Seal Thickness	N/A
Lid Config	N/A	Parts to Render	Reactor
Nitrogen Needle	N/A	Part Quality	High
Suba Size	N/A		
Number Electrode Holes	N/A		
Electrode Hole Shape	N/A		
Electrode Hole Diameter	N/A		
Rectangular Electrode X	N/A		
Rectangular Electrode Y	N/A		
Electrode Separation	N/A		

Table S7. ERCAD parameter settings for the Electrasyn compatible cell pictured in Figure **S7**.

Scaleup of Acetoxylation Reaction

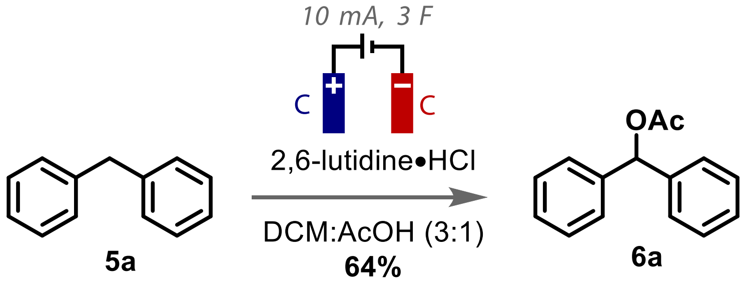

To a 40 mL 3D printed Electrasyn-compatible cell equipped with a stirrer bar was added diphenylmethane (340 mg, 2.0 mmol) and 2,6-lutidine·HCl (788 mg, 4.0 mmol, 2 equiv.). The threaded neck of the cell was wrapped in teflon tape, and the cell sealed with a standard Electrasyn lid fitted with graphite working and counter electrodes. The reaction vessel was purged with nitrogen for 0.25 h, then DCM (30 mL) and glacial acetic acid (10 mL) were added. The reaction mixture was stirred for 0.25 h under an N_2 atmosphere, and then subjected to electrolysis (10 mA, 3.0 F). The reaction mixture was transferred to a conical flask, cooled on ice and quenched by the addition of saturated aqueous $NaHCO_3$ (100 mL). The electrodes were rinsed with DCM into the same flask. The biphasic mixture was transferred to a separating funnel and the layers separated. The organic layer was extracted with $NaHCO_3$ (3 × 50 mL) and water (50 mL), then dried over $MgSO_4$, filtered, and concentrated *in vacuo*. Purification by silica column chromatography (20:1 Pentane:Diethyl Ether) afforded the desired benzhydryl acetate **6** as a colourless oil (294 mg, 64%).

The following data are in accordance with those previously reported.[5]

Benzhydryl acetate

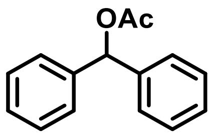

6a

^{1}H NMR (400 MHz, $CDCl_3$) δ: 7.41-7.28 (10H, m, Ar-H), 6.92 (1H, s, CH), 2.18 (3H, s, CH_3)

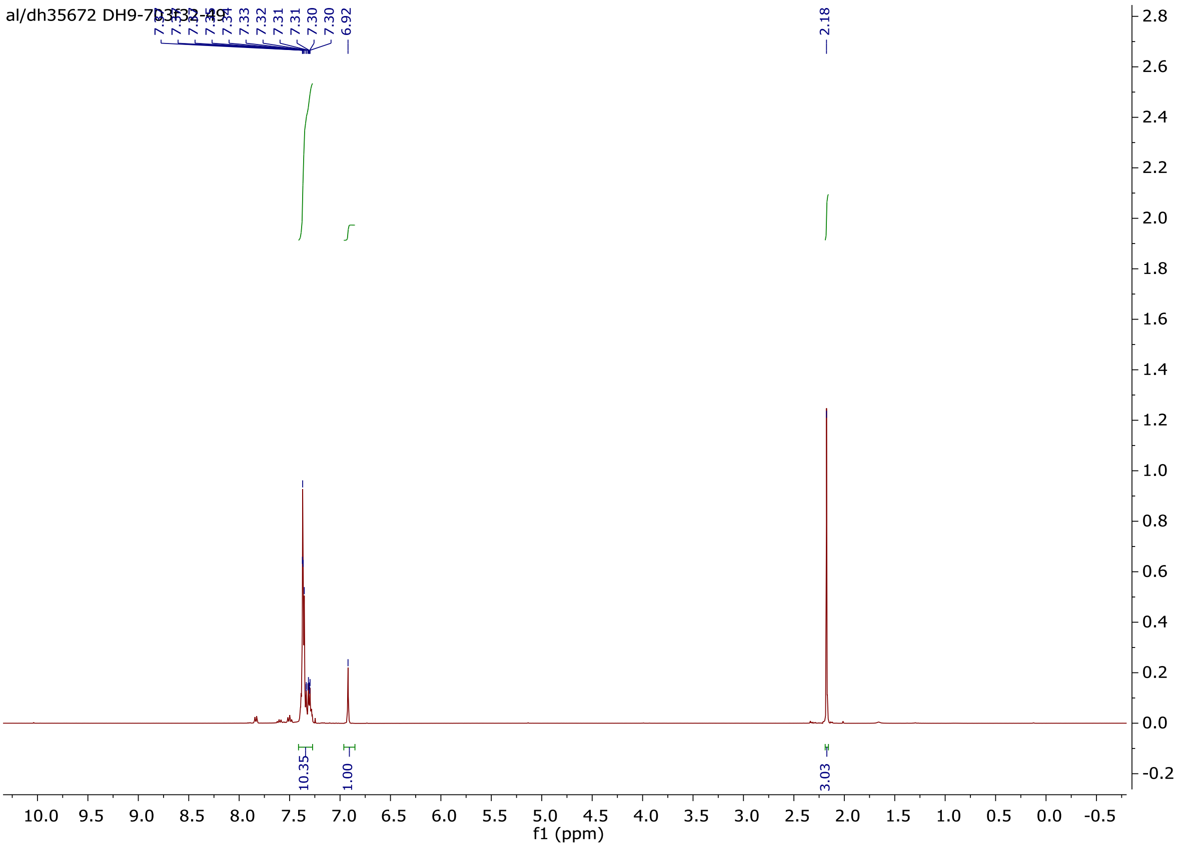

^{13}C NMR (101 MHz, CDCl$_3$) δ: 170.1 (C=O), 140.3 (Ar), 128.6 (Ar), 128.0 (Ar), 127.2 (Ar), 77.0 (C-O), 21.4 (CH_3).

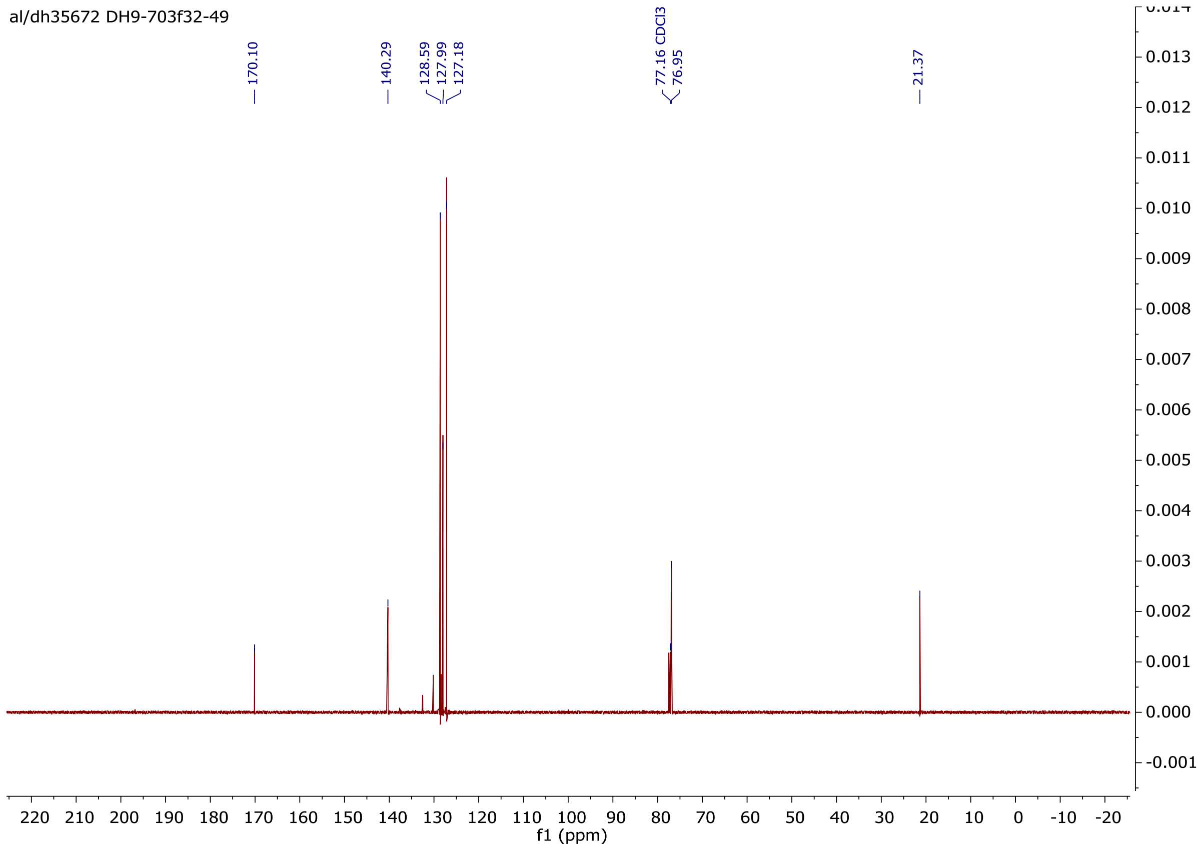

Screening Unit – Acetoxylation/Acyloxylation Reaction

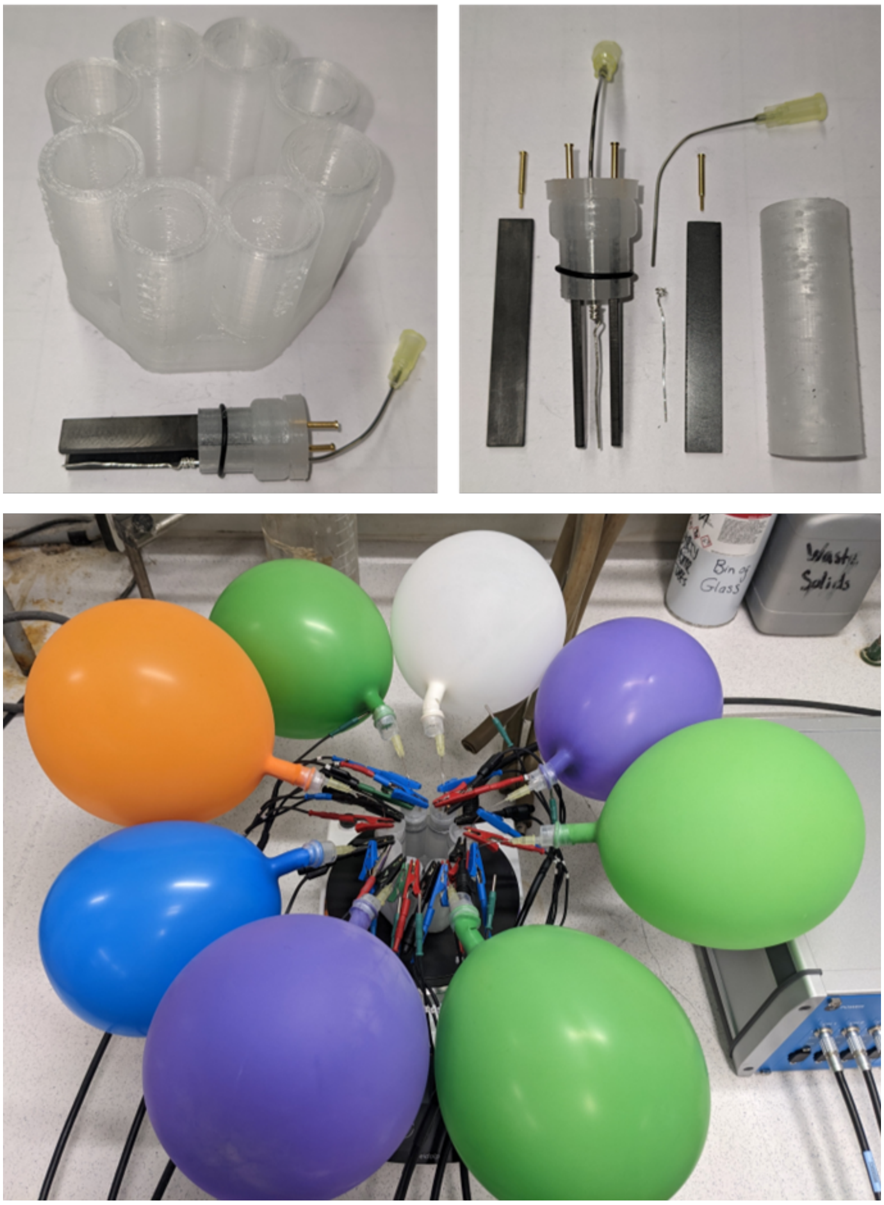

Figure S8. 8 × 8 mL 3D printed screening setup reactor used for benzylic acetoxylation and acyloxylation. Airtight lid assembly shown below.

Parameter	Value	Parameter	Value
**Reactor Type**		**Reference Electrode**	
Divided or Undivided?	Undivided	Ref Electrode	N/A
Electrasyn Compatible?	No	Ref Electrode Diameter	N/A
Number of Reactors	8	Ref Separation	N/A
Label	"Screening Setup"		
**Reactor Body Parameters**		**Divided Cell Assembly**	
VolumeML	8	Bolt Size	N/A
Wall Thickness	3	Divider Diameter	N/A
Base Thickness	3	Frit Thickness	N/A
Base Curvature	50	Dividing Wall Thickness	N/A
**Reactor Lid Parameters**		**Other Parameters**	
Push Fit	Yes	HR Ratio	4
Lid Seal	Yes	Seal Thickness	2
Lid Config	Electrodes Only	Parts to Render	Lid & Reactor
Nitrogen Needle	Yes	Part Quality	High
Suba Size	N/A		
Number Electrode Holes	2		
Electrode Hole Shape	Electrasyn		
Electrode Hole Diameter	N/A		
Rectangular Electrode X	N/A		
Rectangular Electrode Y	N/A		
Electrode Separation	2-6		

Table S8. ERCAD parameter settings for the 8x8 mL screening setup pictured in Figure **S8**

Screening Setup - Airtight Lid Assembly

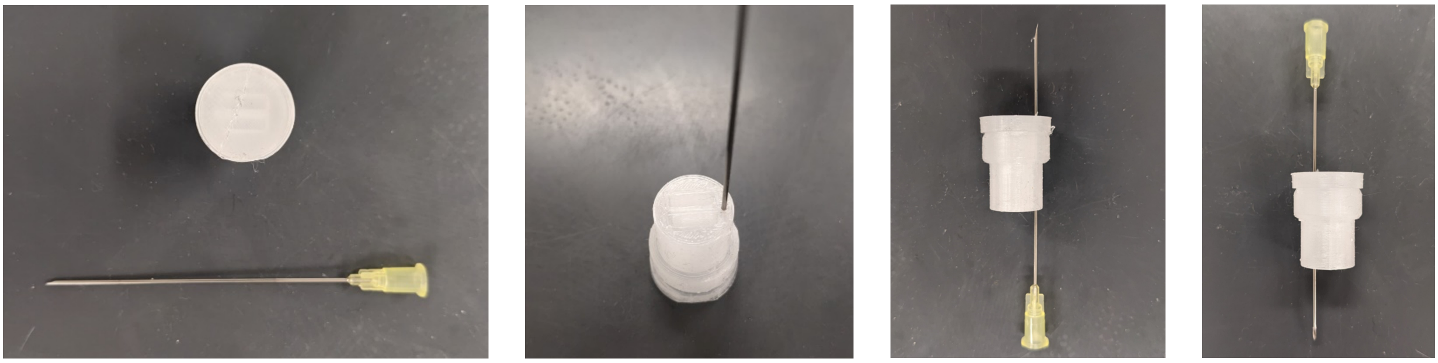

1) Using a 21g needle, pierce the polypropylene layer covering the nitrogen inlet.

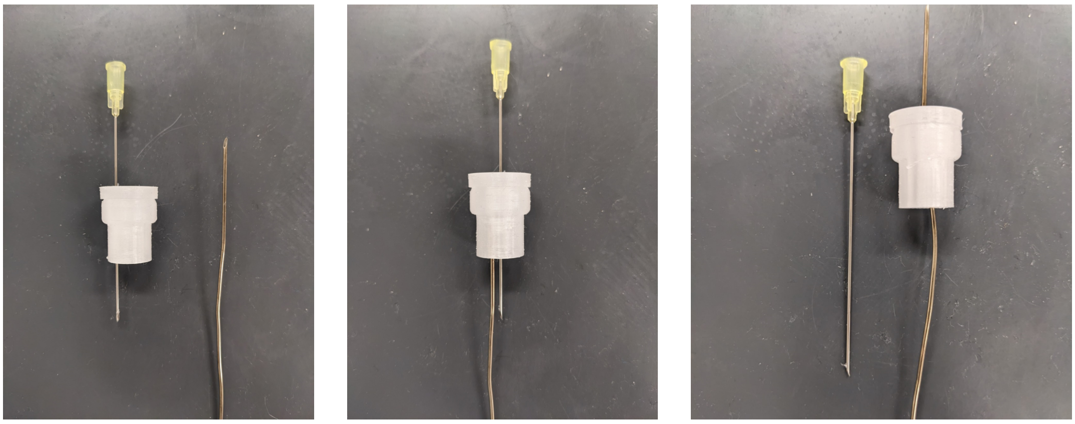

2) Using a 21 g reusable needle, pierce the polypropylene layer covering the electrode hole, repeat for other electrode.

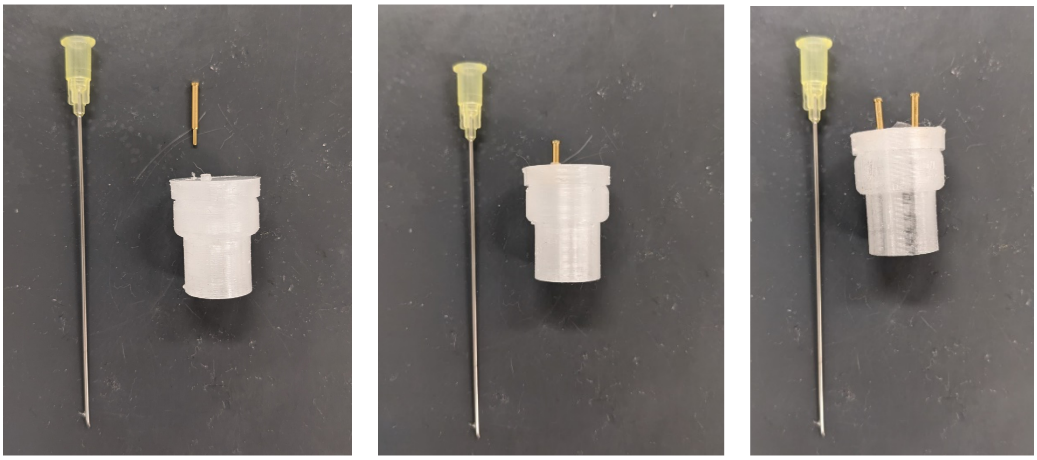

3) Insert two spring loaded test pin probes.

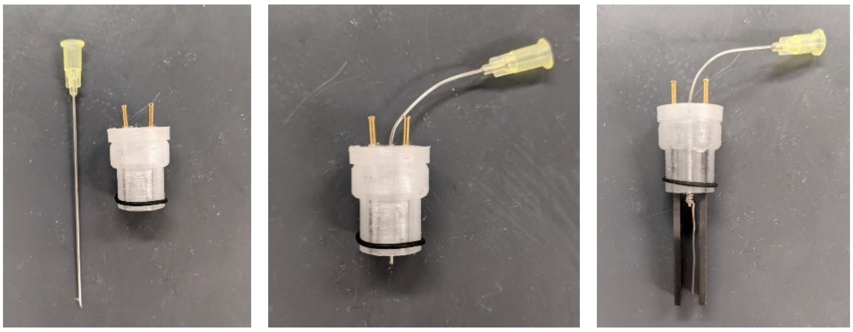

4) Affix O-ring, insert nitrogen inlet and attach silver psuedo reference wire if needed.

For the alternative method of installing heat set inserts into the 3D printed lid using a soldering iron, see this video for more information on an example of this process: https://www.youtube.com/watch?v=P7nHyI1TwKY

General Procedure for Acetoxylation Reaction

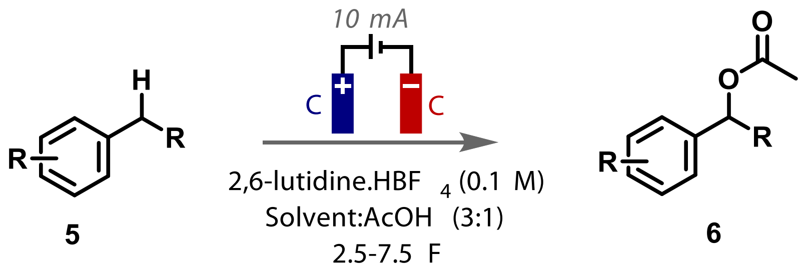

To the 8 × 8 mL 3D printed screening setup reactor that had been dried in a vacuum oven (80 °C) overnight and equipped with magnetic stirrer bars was added substrate (0.2 mmol, 1 equiv.) and 2,6-lutidine•HBF_4 (0.40 mmol, 2.0 equiv., 77.8 mg). Solvent (3.0 mL) and glacial acetic acid (1.0 mL) were then added. The reactors were then capped and sealed with push fit lids bearing nitrogen balloons, silver wire pseudo reference electrode and graphite working and counter electrodes (electrode separation = 2, 4, or 6 mm). The reaction mixture was subjected to electrolysis with a Palmsens potentiostat (10 mA constant current for 2.5 - 7.5 F). Upon completion, a known quantity of ^{1}H NMR standard (1,3,5-trimethoxybenzene) was added.

^{1}H NMR yields were attained because assessments and comparisons of the reactors were the primary focus. An aliquot of the reaction mixture was dissolved in d_6-DMSO and the amount of product formed was determined by ^{1}H NMR from the ratio of the benzylic product peak known from literature[5] to the ^{1}H NMR standard (6.08 ppm (3H, s)). Due to the higher resistance of EtOAc, reactions run with this solvent may have over exceeded the compliance voltage of the instrument, and therefore the charge passed may have been lower than expected.

Benzhydryl acetate

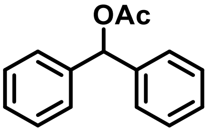

6a

Title compound **6a** was prepared from diphenylmethane following the general procedure for acetoxylation reaction experimental using propylene carbonate and an electrode separation of 6 mm. A recorded NMR yield of 77%, after 3 F, was obtained. The amount of product formed was determined by ^{1}H NMR from the ratio of the characteristic benzylic product peak (δ = 6.74 (1H, s)) to 1 equivalent of 1,3,5-trimethoxybenzene (δ = 6.05 (3H, s)).

The characteristic product peak is in agreement with the literature (δ = 6.89 (s, 1H)).[5]

^{1}H NMR (400 MHz, d_6-DMSO)

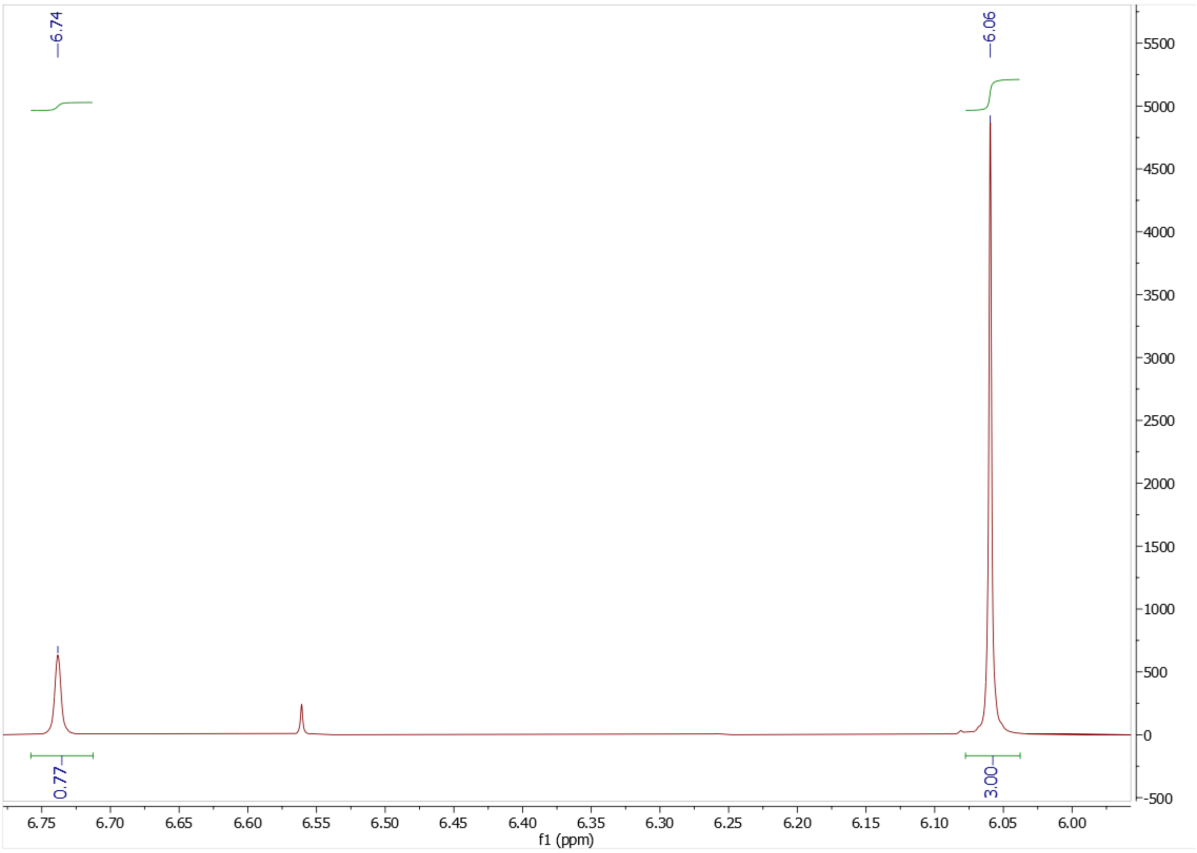

1-(3-(*tert*-butyl)phenyl)ethyl acetate

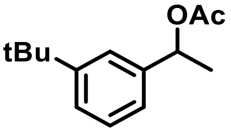

6b

Title compound **6b** was prepared from 1-(*tert*-butyl)-3-ethylbenzene following the general procedure for acetoxylation reaction experimental using propylene carbonate and an electrode separation of 6 mm. A recorded NMR yield of 79%, after 2.5 F, was obtained. The amount of product formed was determined by ^{1}H NMR from the ratio of the characteristic benzylic product peak (δ = 5.79 (1H, q)) to 1 equivalent of 1,3,5-trimethoxybenzene (δ = 6.08 (3H, s)).

The characteristic product peak is in agreement with the literature (δ = 5.90 (q, J = 6.6 Hz, 1H)).[5]

^{1}H NMR (400 MHz, d_6-DMSO)

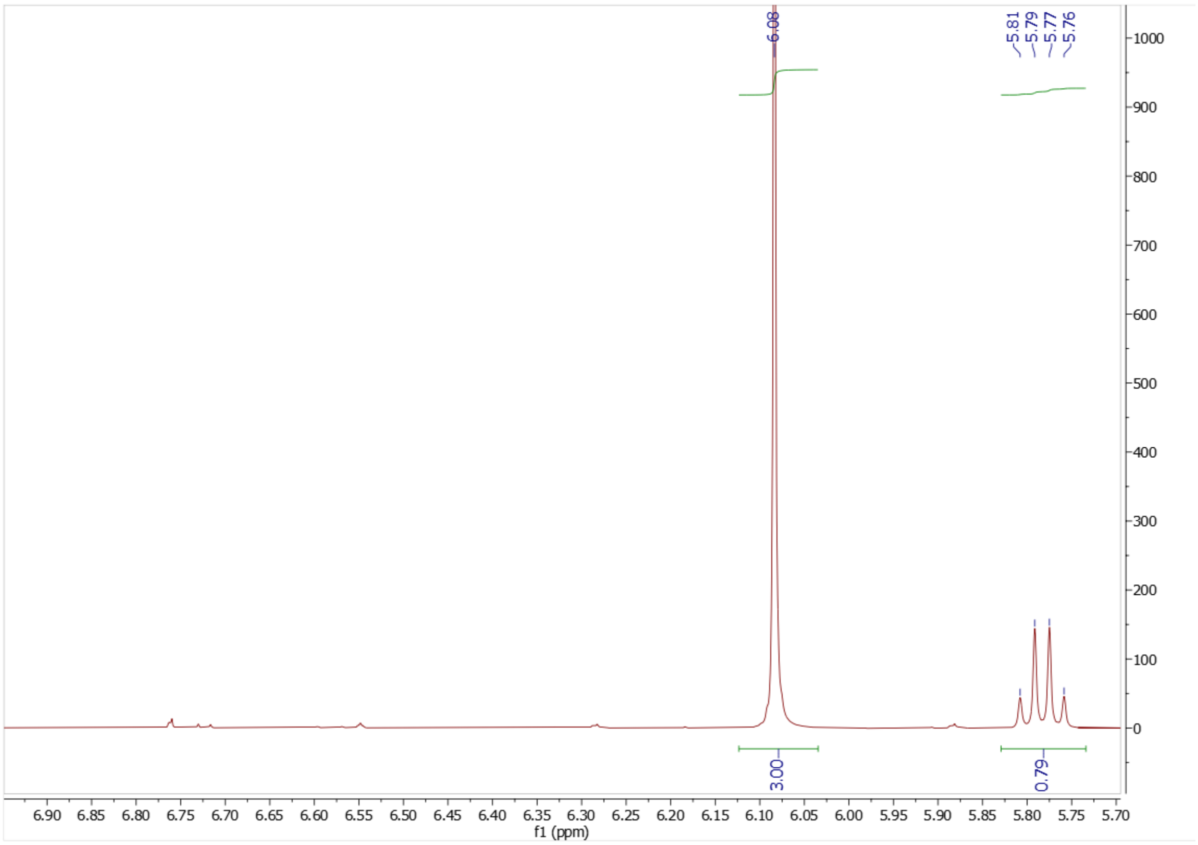

1-(4-Bromophenyl)ethyl acetate

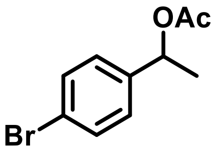

6c

Title compound **6c** was prepared from 1-bromo-4-ethylbenzene following the general procedure for acetoxylation reaction experimental using propylene carbonate and an electrode separation of 6 mm. A recorded NMR yield of 57%, after 3 F, was obtained. The amount of product formed was determined by ^{1}H NMR from the ratio of the characteristic benzylic product peak (δ = 5.74 (1H, q)) to 1 equivelent of 1,3,5-trimethoxybenzene (δ = 6.06 (3H, s)).

The characteristic product peak is in agreement with the literature (δ = 5.82 (q, J = 6.6 Hz, 1H)).[5]

^{1}H NMR (400 MHz, d_6-DMSO)

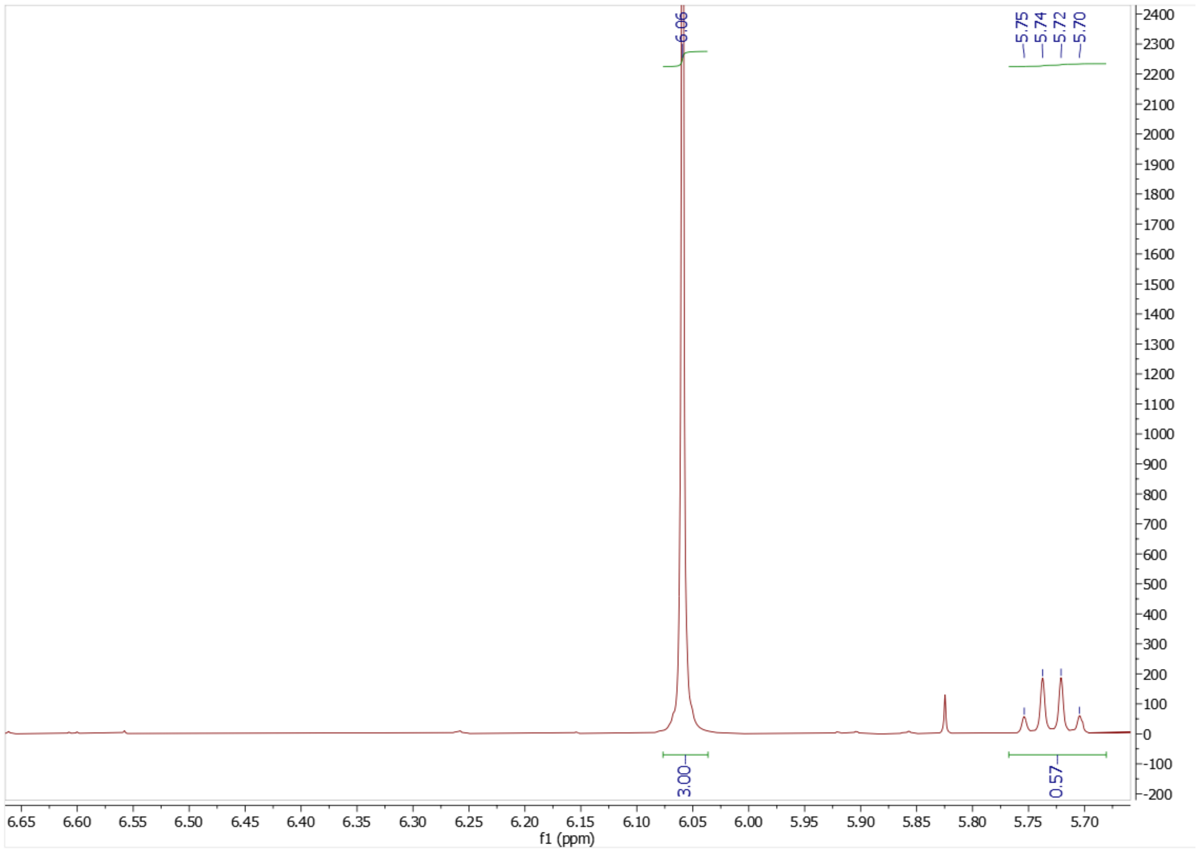

1-Phenylethyl acetate

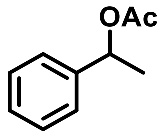

6d

Title compound **6d** was prepared from ethylbenzene following the general procedure for acetoxylation reaction experimental using propylene carbonate and an electrode separation of 6 mm. A recorded NMR yield of 70%, after 3 F, was obatined. The amount of product formed was determined by ^{1}H NMR from the ratio of the characteristic benzylic product peak (δ = 5.76 (1H, q)) to 1 equivalent of 1,3,5-trimethoxybenzene (δ = 6.07 (3H, s)).

The characteristic product peak is in agreement with the literature (δ = 5.89 (q, J = 6.6 Hz, 1H)).[5]

^{1}H NMR (400 MHz, d_6-DMSO)

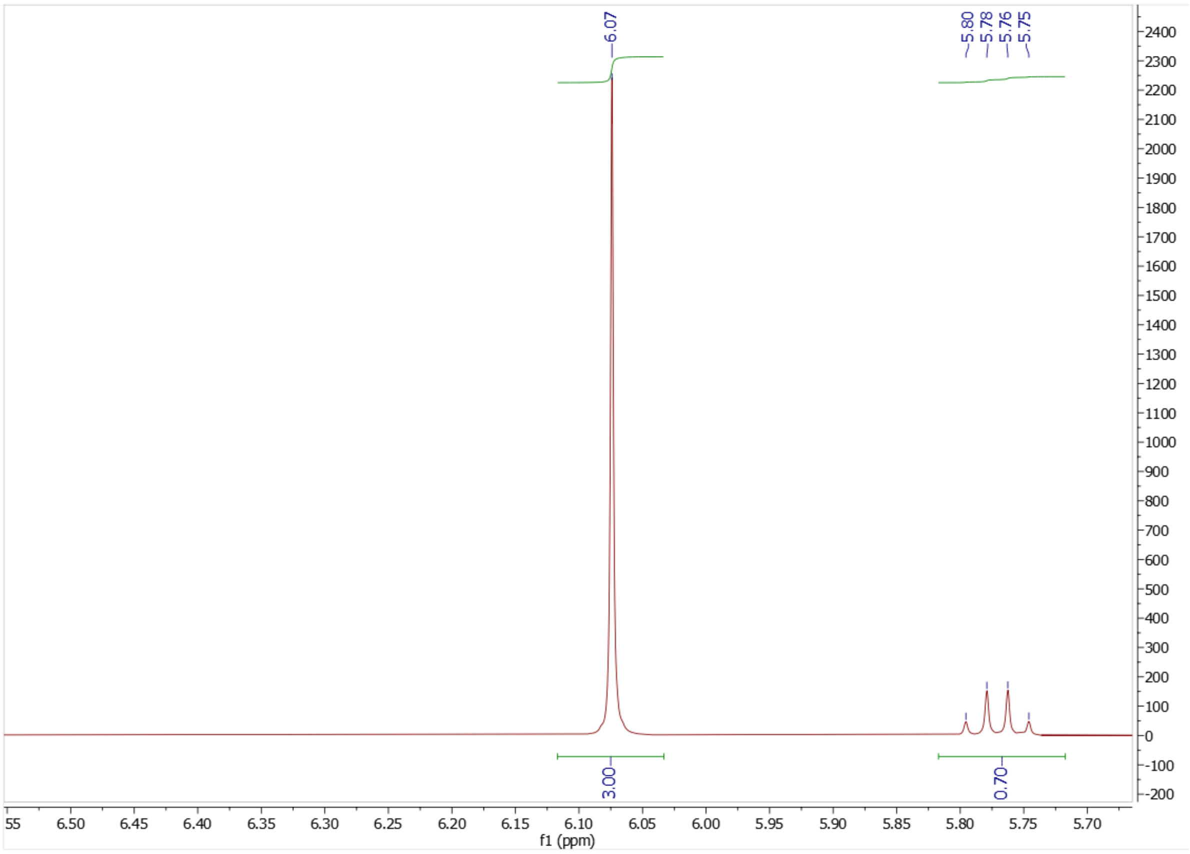

1-(4-Bromophenyl)pentyl acetate

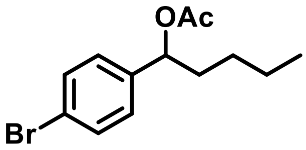

6e

Title compound **6e** was prepared from 1-bromo-4-pentylbenzene following the general procedure for acetoxylation reaction experimental using propylene carbonate and an electrode separation of 6 mm. A recorded NMR yield of 81%, after 2.5 F, was obtained. The amount of product formed was determined by ^{1}H NMR from the ratio of the characteristic benzylic product peak (δ = 5.60 (1H, appt)) to 1 equivalent of 1,3,5-trimethoxybenzene (δ = 6.07 (3H, s)).

The characteristic product peak is in agreement with the literature (δ = 5.65 (dd, J = 7.5, 6.5 Hz, 1H)).[5]

^{1}H NMR (400 MHz, d_6-DMSO)

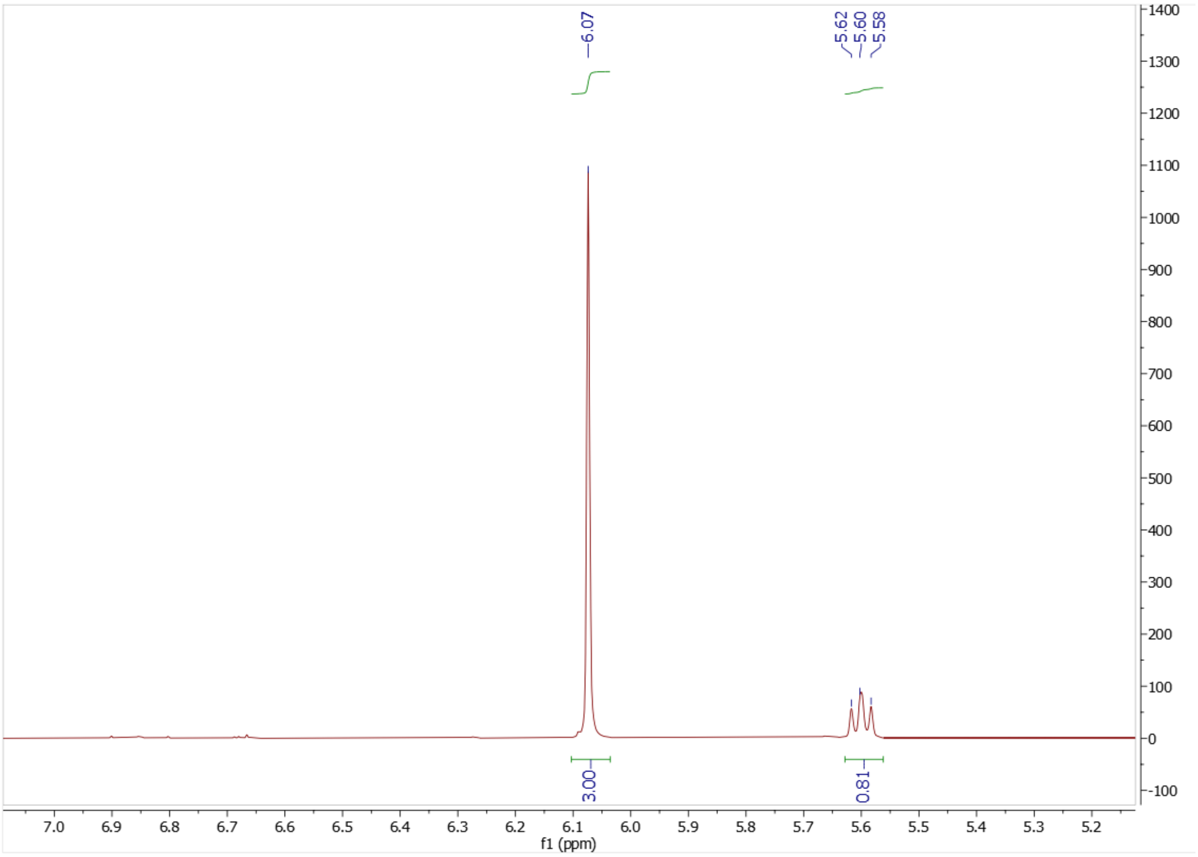

1,2,3,4-Tetrahydronaphthalen-1-yl acetate

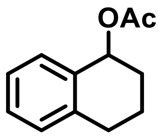

6f

Title compound **6f** was prepared from 1,2,3,4-tetrahydronaphthalene following the general procedure for acetoxylation reaction experimental using propylene carbonate and an electrode separation of 6 mm. A recorded NMR yield of 71%, after 3 F, was obtained. The amount of product formed was determined by ^{1}H NMR from the ratio of the characteristic benzylic product peak (δ = 5.86 (1H, t)) to 1 equivalent of 1,3,5-trimethoxybenzene (δ = 6.07 (3H, s)).

The characteristic product peak is in agreement with the literature (δ = 6.01 (t, J = 4.3 Hz, 1H)).[5]

^{1}H NMR (400 MHz, d_6-DMSO)

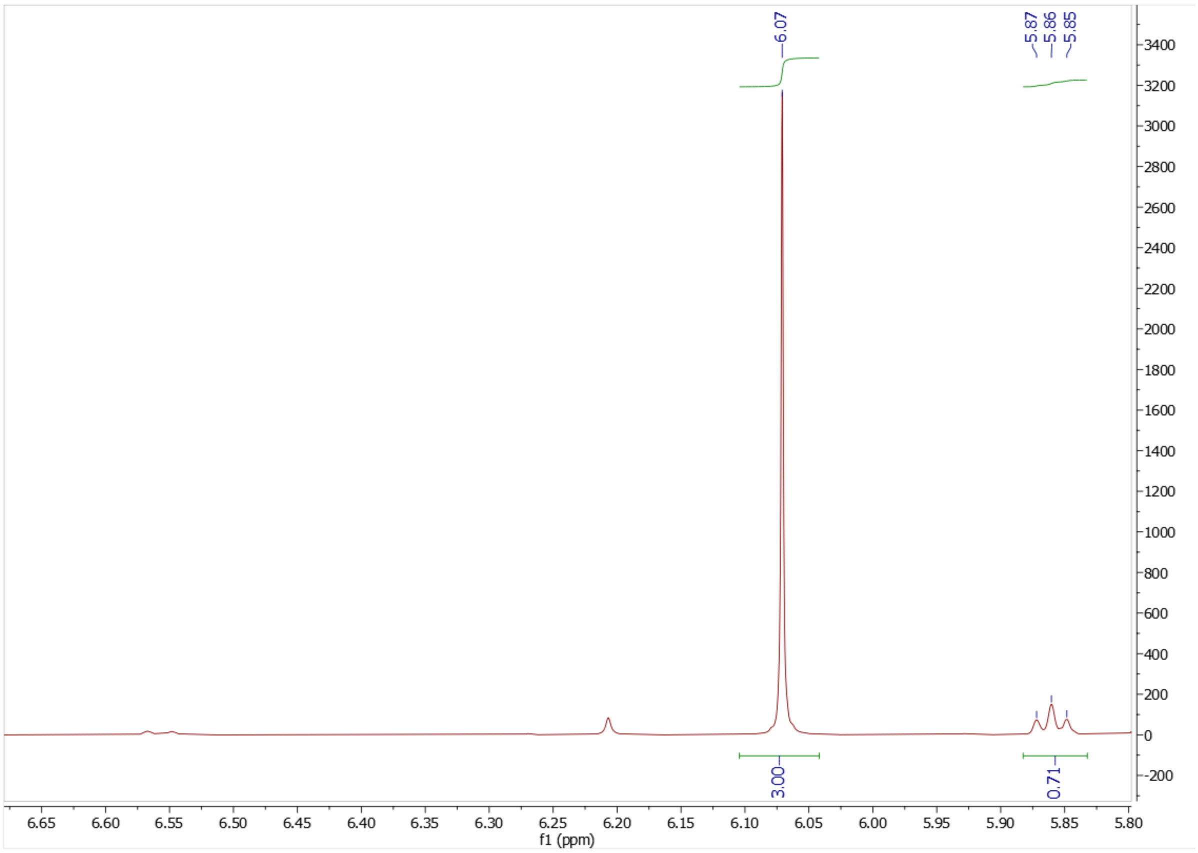

Acyloxylation of 1-*tert*-butyl-3-ethylbenzene

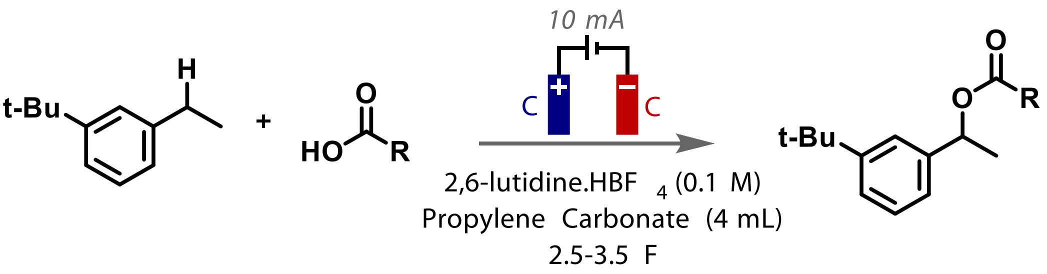

To an 8 × 8 mL 3D printed screening setup that had been dried in a vacuum oven (80 °C) overnight and equipped with magnetic stirrer bars was added 1-*tert*-butyl-3-ethylbenzene (0.2 mmol, 1 equiv.) and 2,6-lutidine•HBF_4 (0.40 mmol, 2.0 equiv., 77.8 mg). Then were added propylene carbonate (4.0 mL) and acid (0.6-2.0 mmol, 3-10 equiv.). The reactors were then capped and sealed with push fit lids bearing nitrogen balloons, silver wire pseudo reference electrode and graphite working and counter electrodes (electrode separation = 6 mm). The reaction mixture was subjected to electrolysis with a Palmsens potentiostat (10 mA constant current for 2.5-7.5 F). Upon completion, a known quantity of NMR standard (1,3,5-trimethoxybenzene) was added.

^{1}H NMR yields were attained because assessments and comparisons of the reactors were the primary focus. An aliquot of the reaction mixture was dissolved in d_6-DMSO and the amount of product formed was determined by ^{1}H NMR from the ratio of the characteristic benzylic product peak known from literature[5] to the NMR standard (6.08 ppm (3H, s)).

1-(3-(*tert*-Butyl)phenyl)ethyl 4-fluorobenzoate

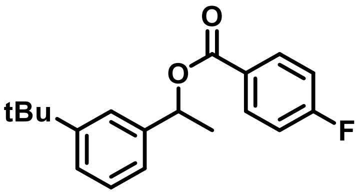

6g

Title compound **6g** was prepared from 3 equivalents of 4-fluorobenzoic acid following the acyloxylation of 1-(*tert*-butyl)-3-ethylbenzene experimental with a recorded NMR yield of 72%, after 3 F. The amount of product formed was determined by ^{19}F NMR from the ratio of the characteristic fluorine product peak (δ = -106.32 (1F, m)) to 1 equivalent of ethyl fluoroacetate (δ = -230.06 (1F, t)).

The characteristic product peak is in agreement with the literature (δ = -105.8 (tt, J = 8.4, 5.4 Hz).).[5]

19^{F} NMR (376 MHz, d_6-DMSO)

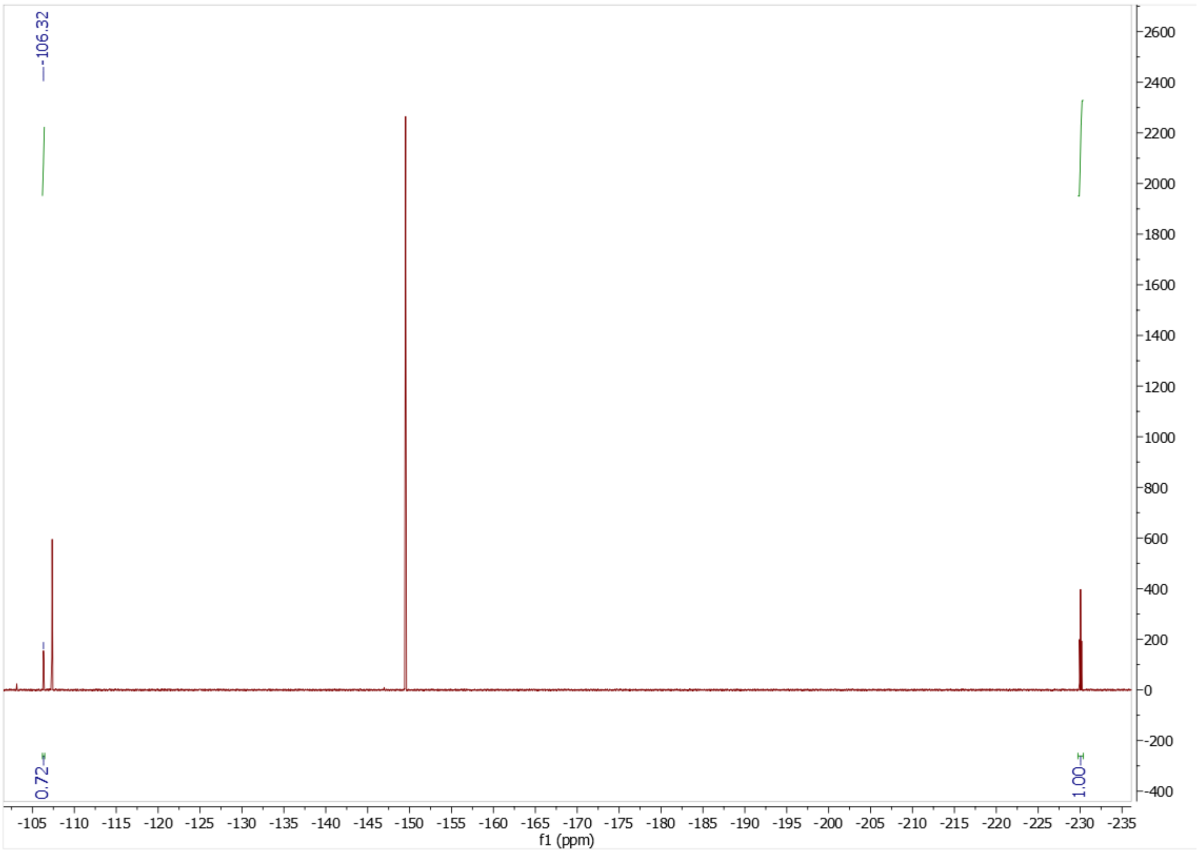

1-(3-(*tert*-Butyl)phenyl)ethyl benzoate

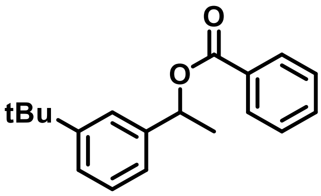

6h

Title compound **6h** was prepared from 10 equivalents of benzoic acid following the acyloxylation of 1-(*tert*-butyl)-3-ethylbenzene experimental with a recorded NMR yield of 98%, after 2.5 F. The amount of product formed was determined by ^{1}H NMR from the ratio of the characteristic benzylic product peak (δ = 6.13 (1H, q)) to 1 equivalent of 1,3,5-trimethoxybenzene (δ = 6.07 (3H, s)).

The characteristic product peak is in agreement with the literature (δ = 6.16 (q, J = 6.6 Hz, 1H)).[5]

^{1}H NMR (400 MHz, d_6-DMSO)

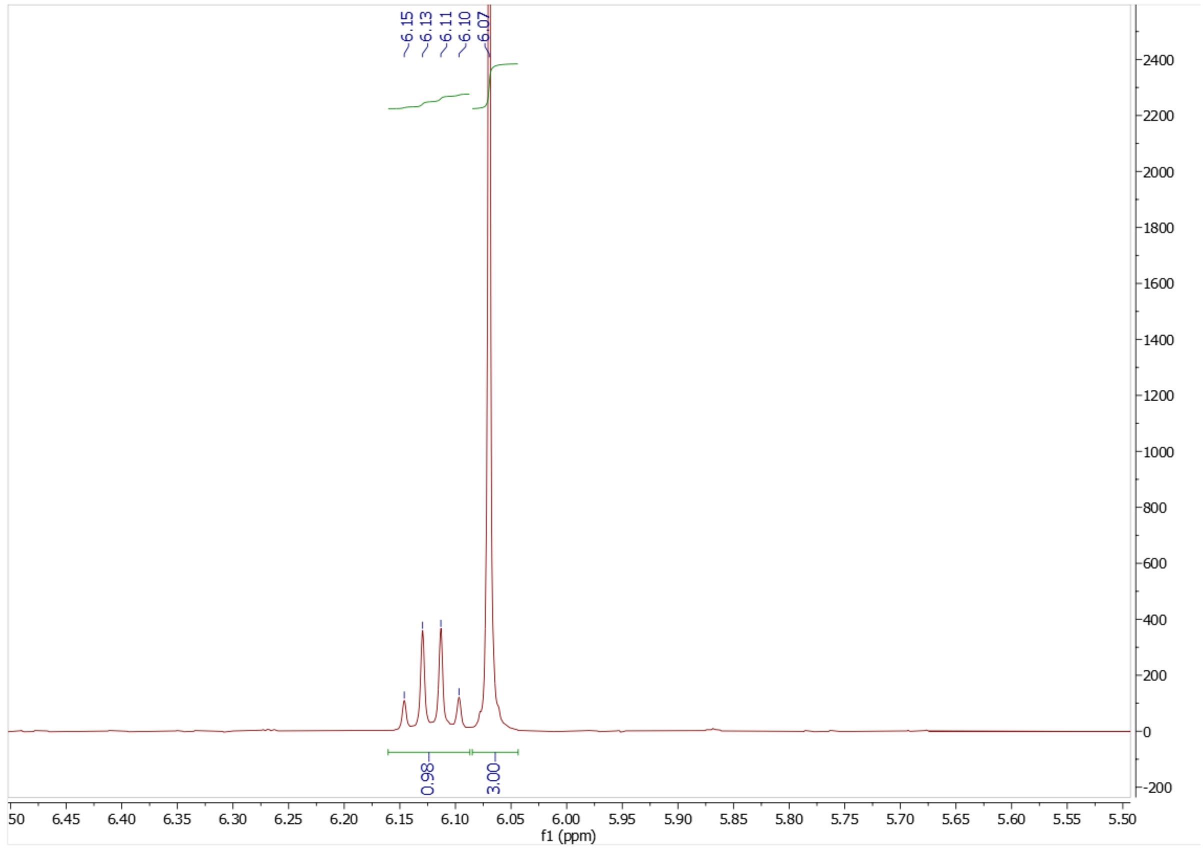

Code Appendix

The OpenSCAD code for ERCAD is given below, however a .scad file containing the code, a .json file containing the reactor parameters required to produce the reactors used in this study, and .stl files for each reactor, are available at data.bris, at https://doi.org/10.5523/bris.2ko5vmzjsp1z1290n89glwgz3r.

ERCAD

```
/*ERCAD: Electrochemical Reactor Computer Aided Design

To generate a reactor, please select your parameters from the customiser tab (this may
need to be enabled in OpenSCAD if not visible, Check Window>>Hide Customiser is
unchecked). You can save each design as a Preset using the options at the top of the
Customiser window. These are saved as a .json file in the same folder as this file is saved.

ERCAD renders each component of a reactor separately. The final option in the customiser
tool is to decide which part to generate an STL file for. After completing your design, press
F6 to render it. Then you can save the STL using the option above. You'll need to repeat this
for every reactor body and lid required.

If you are familiar with OpenSCAD, the rest of this file can be further modified if necessary.
Otherwise, it is suggested that the editor window is not altered.

SAFETY NOTE:
It is possible to generate designs which will not function when printed. Please double check
designs before printing, and any printed piece should be leak-tested before use.
---------------------------------------------------------
*/

/*---------------------------------------------------
USER DEFINED VARIABLES */


/* [Reactor Type] */

//Divided or Undivided?
Compartments="Undivided";//[Divided,Undivided]

//Select whether reactor should fit Electrasyn
Electrasyn_Compatibility=false;

//Number of Joined Reactors (allows screening platforms, not compatible with the
electrasyn)
Number_Reactors=1;//[1,2,3,4,5,6,7,8]

//Enter ID text to print onto reactor (Leaving blank will autogenerate an appropriate label. 8
characters or fewer will fit onto most reactors)
Label="LLab";

/* [Reactor Body Parameters] */

//Select usable volume of reactor
```

```
VolumeML=40;
//Thickness of Reactor Walls (3mm suggested)
Wall_Thickness=3; //0.1
//Thickness of Reactor Base (3mm suggested)
Base_Thickness=3.00; //0.1
//Percentage of curvature
Base_Curvature=50; // [1:100]

/* [Reactor Lid Parameters] */

//Check this box to generate a push-fit lid, rather than a screw-fit. Use this in combination with a lid seal (option below) in order to generate a sealable reactor.
Push_Fit=false;

//The lid will be sealed with a thin layer of plastic which can be pierced, in order to improve how air-tight the reactor is. For advice on achieving this, refer to the SI.
Lid_Seal=false;

//Types of holes required in the reactor lid.
Lid_Config="Electrodes Only";//[Electrodes Only,Electrodes & Suba, Suba Only]

//Include needle inlet for nitrogen?
Nitrogen_Needle=false;

//Size of Suba seal to use (0.5 mm added to true diameter for tolerance)
Suba_Size=12;//[5.25:B10, 7.25:B14, 8.75:B19, 12.25:B24]

//Number of holes in each reactor lid for electrodes
Number_Electrode_Holes=1;//[0,1,2,3:3 Linear,4:3 Equidistant, 5:2 + Reference]
//Shape of the Electrode Holes
Electrode_Hole_Shape="Circular";//[Circular,Square,Rectangle,Electrasyn]
//Diameter of Circular or Square Electrode Holes (mm)
Electrode_Hole_Size=5; //0.01
//Length of Short Edge of Rectangular Electrode (mm)
Rectangular_Electrode_X=1; //0.1
//Length of Long Edge of Rectangular Electrode (mm)
Rectangular_Electrode_Y=3; //0.1
//Distance between electrodes (mm)
Electrode_Separation=5; //0.1

/* [Reference Electrode] */

//Would you like to select separate dimensions for a reference electrode
Ref_Electrode=false;
Ref_Electrode_Diameter=6; //0.01
Ref_Separation=5; //0.1


/* [Divided Cell Assembly] */
//Size of Bolts used to assemble reactor (M4 works well, however arrays of small reactors may require a smaller size to render correctly)
Bolt_Size=4;//[1:M1,2:M2,3:M3,4:M4]
//Diameter of glass frit+O-ring used to divide compartments (mm). The glass frit should be of a thickness slightly less than the O-ring surrounding it.
Divider_Diameter=20;
```

```
//Thickness of the glass frit+O-ring combination (mm). If more than 2mm, may need to
increase the dividing wall thickness.
Frit_Thickness=2;
//Dividing Wall Thickness (mm) (per reactor, will be double for assembled cell. Note wall
thickness will also increase this distance. Electrasyn Electrodes have a 6mm electrode
separation which must be respected for Electrasyn-compatible designs.
Dividing_Wall_Thickness=2;

/* [Other Parameters] */

//Height-to-Radius Ratio of the reactor (default is 4, cannot be <1. Applies to non-electrasyn
designs)
HR_Ratio=4;
//Thickness of sealing layer applied to top of the lid
Seal_Thickness=4;


/* [Choose a part to render (F6):] */
Part="Undivided Reactor";//[Undivided Reactor, Divided Part A, Divided Part B, Lid]
//Choose the quality of the part to generate (determines render speed)
Part_Quality=1;//[1:Low,2:Medium,3:High]
/*----------------------------------------------------
OTHER VARIABLES */

 /* [Hidden] */

Actual_Volume=VolumeML*1000; // Calculates volume in L not mL
Curve_Points=40*Part_Quality; //$fa and $fs also defined
$fa=(6/Part_Quality);
$fs=(0.25/Part_Quality);
Internal_Radius_Reactor=pow((Actual_Volume/((HR_Ratio)*PI)),1/3);
Seal_Thickness_NonUser = Lid_Seal==true ? Seal_Thickness: 0; //Adjust this value to
change the thickness of the sealing plastic applied to the top of the lid.

Height_Reactor= Electrasyn_Compatibility==true ? 65 : HR_Ratio*Internal_Radius_Reactor;
Internal_Radius_Esyn=pow((Actual_Volume/(PI*(Height_Reactor-Base_Thickness-11-
10))),1/2); //-Base_Thickness-10-10 accounts for Base Thickness, screw thread and
expansion region. Reactor volume is therefore straight-sided section as with non-Esyn
reactors.

Degree_Curvature=0.01*(Base_Curvature-1)*Internal_Radius_Reactor;
Degree_Curvature_Esyn=0.01*(Base_Curvature-1)*Internal_Radius_Esyn;

Divider_Width=(2*Internal_Radius_Reactor)+(2*Wall_Thickness);
Divider_Bolt_Section=3*Bolt_Size;

Electrasyn_Electrode_X=2.2; //Short edge of electrasyn electrode
Electrasyn_Electrode_Y=9; //Long edge of electrasyn electrode

Suba_Height=lookup(Suba_Size,[
	[5.25,19],
	[7.25,23],
	[8.75,26],
	[12.25,29]]);
```

```
Suba_Minor_Radius=0.5*((2*Suba_Size)-(0.1*Suba_Height));
Suba_Offset= Lid_Config=="Electrodes & Suba" ? Suba_Minor_Radius+Wall_Thickness : 0;
Ref_Offset= Ref_Separation+(0.5*Electrode_Hole_Size)+(0.5*Ref_Electrode_Diameter);
Ref_Offset_2=Ref_Separation+(0.5*Ref_Electrode_Diameter)+(0.5*Rectangular_Electrode_
Y);
Ref_Offset_3=Ref_Separation+(0.5*Ref_Electrode_Diameter)+(0.5*Electrasyn_Electrode_Y
);

t_d1 = Push_Fit==true ? 0: 2.36;  // Diameter of coils in the helix (2.36 for GL25 Electrasyn)
t_d2 = (2*Internal_Radius_Reactor); // Diameter of central thread (add d1 for max diameter )
t_h1 = 1.14; // Height between coils (0 means coils have no separation)
t_h2 = 10; // Height of total coil (should be non-zero)

t_d1_Esyn=2.36;
t_d2_Esyn=22.5;
t_h1_Esyn=1.14;
t_h2_Esyn=10;
Esyn_Screw= Electrasyn_Compatibility==true ? 0: 1; //NOTE: Leaving Electrasyn-
compatibility checked when designing lids adds extra screw thread to them which will not
necessarily match printed undivided reactors. Electrasyn compatible lids are not a feature of
ERCAD.

//Translation Distance sets how far from the centre each reactor should be moved, to
assemble a monolith
Translation_Distance=(((2*Internal_Radius_Reactor+2*Wall_Thickness-(Wall_Thickness-
0.5*t_d1))/2)/sin(360/(2*Number_Reactors)))+(Wall_Thickness-(0.5*t_d1));

//Below tests whether a label has been added. If label field is not blank, text is the reactor
number + LL + reactor volume + mL.
Reactor_Text= Label!="" ? Label: str("LL",VolumeML,"mL");



/*-----------------------------------------------------
SCREW THREAD CODE */

//The code in this section is an iteration upon an original design authored by psync for
Parameterized thread rod, available from https://www.thingiverse.com/thing:5013 , and
following the terms of the creative commons license.

fn_value = 16; // $fn value for torus, makes it look
                                        // smoother the higher the value. Note, increasing this value
will give smoother walls of the reactor, but this has a significant impact on render time.

// These values match the Electrasyn and are provided for reference.
// t_d1 = 2.36;  // Diameter of coils in the helix
// t_d2 = 22.5; // Diameter of central thread (add d1 for max diameter)
// t_h1 = 1.14; // Height between coils (0 means coils have no separation)
// t_h2 = 10; // Height of total coil (should be non-zero)
// $fn=80;

module half_torus( d1, w )
{
        rotate_extrude(convexity = 10, $fn=fn_value*2)
                translate([w/2,0,0])
```

```
                circle(r=d1/2,$fn=fn_value/2);
} // half_torus module //

module basic_helix( d1, w, h1 )
{
    // This piece forms the bottom half of the helix
    rotate( a = -asin((d1+h1)/(2*w)), v=[0,1,0] )
    {
        union()
        {
        translate([w/2,0,0])
            difference()
            {
                half_torus(d1,w);
                // Translate -0.1 extra for manifold
                // intersection
                translate([0,-(w+d1)/4-0.1,0])
                    cube( size = [w+d1,(w+d1)/2,d1],
                        center = true );
            } // end of difference()
        } // end of union()
    } // end of rotate()
} // basic_helix module //

//basic_helix( t_d1, t_d2, t_h1 ); // Debug output checking

module basic_unit( d1, w, h1 )
{
    union()
    {
        // Lay down the first half-torus
        basic_helix( d1, w, h1 );
        // Now mirror it and place a copy above
        translate([0,0,h1+d1])
            mirror([0,1,0])
                mirror([0,0,1])
                basic_helix( d1, w , h1);
    } // end of union()
} // basic_unit module //

//basic_unit( t_d1, t_d2, t_h1 ); // Debug output checking

module main_helix( d1, w, h1, h2 )
{
    union()
    {
        for ( i = [0:(h1+d1):h2] )
        {
        translate([0,0,i])
            basic_unit( d1, w, h1 );
        } // end of for()
    } // end of union()
} // main_helix module //

//main_helix( t_d1, t_d2, t_h1, t_h2 ); // Debug output checking
```

```
module spring( d1, d2, h1, h2 )
{
	// Calcuate w (internal torus width) from d2
	w = sqrt( pow(d2,2) + pow((h1+d1)/2,2) );

	difference()
		{
		// Center the helix
		translate([-d2/2,0,-d1/2])
			main_helix( d1, d2, h1, h2 );

		// Subtract a box to slice off the bottom of the spring (not applied for external
thread on electrasyn thread on reactor body)
		translate([0,0,-d1/2])
			cube(size =
[Esyn_Screw*(d2+2*d1),Esyn_Screw*(d2+2*d1),Esyn_Screw*d1],
				center = true);
		}
} // spring module //

// spring( t_d1, t_d2, t_h1, t_h2 ); // Debug output checking

module screw_thread( d1, d2, h1, h2 )
{
	// Calculate the base diameter, edge to edge
	//w = sqrt( pow(d2,2) + pow((h1+d1)/2,2) );
	mirror([0,1,0]){
	difference() // Subtract the top slice
	{
		union()
		{
			// Main helical spring
			spring( d1, d2, h1, h2 );

			// Central thread cylinder
			translate( [0,0,h2/2] )
				cylinder( h = h2, r = d2/2, center = true, $fn=2*fn_value);
		} // end of union()

		// Top slicing box - uncomment this section to have a flat top profile to threads
		/*

      translate([0,0,h2+(h2+h1)/2])
			cube( size = [ d2+2*d1, d2+2*d1, h2+h1],
				center = true );

      */

	}
} // end of difference()
} // screw_thread module //

//mirror([0,1,0])screw_thread( t_d1, t_d2, t_h1, t_h2 ); // This line produces the Electrasyn
appropriate thread. Comment this out if you want to
```

```
                                                            // call this module
externally

// translate([0,0,-10])cylinder(h=10, r=12.5, center=false);




/*--------------------------------------------------
MODULE CODE */

//Base fillet codes
module Internal_Reactor_Fillet(){
rotate_extrude(angle=360){
translate([Internal_Radius_Reactor-Degree_Curvature,0,0])difference(){
   square([Degree_Curvature,Degree_Curvature], center=false);
   translate([0,Degree_Curvature,0])circle(r=Degree_Curvature);
}
}
}

module Internal_Reactor_Fillet_Esyn(){
rotate_extrude(angle=360){
translate([Internal_Radius_Esyn-Degree_Curvature_Esyn,0,0])difference(){
   square([Degree_Curvature_Esyn,Degree_Curvature_Esyn], center=false);
   translate([0,Degree_Curvature_Esyn,0])circle(r=Degree_Curvature_Esyn,
$fn=Curve_Points);
}
}
}



//The following module creates a basic undivided cell from side walls, a base section, and a
screw threaded section.
module Undivided(){


//Side Wall Section
   linear_extrude(height=Height_Reactor+0.2, center=false){
      difference(){
         circle(r=Wall_Thickness+Internal_Radius_Reactor);
         circle(r=Internal_Radius_Reactor);
      }
   }

//Reactor Base Section

   translate([0,0,-Base_Thickness]){
      cylinder(h=Base_Thickness, r=Wall_Thickness+Internal_Radius_Reactor,
center=false);
   }

//Base Fillet
```

```
    translate([0,0,0])Internal_Reactor_Fillet();

//Reaction Screw Thread Section

    translate([0,0,Height_Reactor])
        difference(){
            cylinder(r=Wall_Thickness+Internal_Radius_Reactor, h=10, center=false);
        // cylinder(r=Internal_Radius_Reactor, h=10, center=false, $fn=Curve_Points);
            screw_thread(t_d1, t_d2, t_h1, t_h2+0.1);
            translate([0,0,-1])cylinder(r=Internal_Radius_Reactor, h=2, center=false);
    }
}

//The following module creates a basic screw-thread lid.
module Lid() {
  //  translate([0,0,Height_Reactor+20])
    //Uncomment above line if wanting to display lid on top of reactor
        //Next block of code creates the basic screw thread lid with solid top.
        union(){
            screw_thread(t_d1, t_d2, t_h1, t_h2);
            translate([0,0,t_h2-0.01])cylinder(r=Internal_Radius_Reactor,h=3, center=false,
$fn=Curve_Points);
            translate([0,0,t_h2+2+3])
            minkowski(){
                cylinder(r=Internal_Radius_Reactor, h=5, center=false, $fn=Curve_Points);
                sphere(r=2);
            }
      }
  }

//The following module creates a cone shaped for a Suba-Seal.

module Suba_Cone(){
    rotate_extrude(angle=360){

polygon(points=[[Suba_Minor_Radius,0],[Suba_Minor_Radius+Wall_Thickness,0],[Suba_Siz
e+Wall_Thickness,Suba_Height],[Suba_Size,Suba_Height]]);

translate([Suba_Size+(0.5*Wall_Thickness),Suba_Height,0])circle(r=0.5*Wall_Thickness,
$fn=Curve_Points);
        translate([Suba_Minor_Radius+Wall_Thickness,0,0])difference(){
            square([3,3], center=false);
            translate([3,3,0])circle(r=3, $fn=Curve_Points);
        }

    }
}

//The following module subtracts electrode shapes from the lid to make the required holes
module Electrode_Lid() {
  union() {
    translate([0,0,t_h2-
0.01+3+5+2+2])cylinder(r=Internal_Radius_Reactor,h=Seal_Thickness_NonUser,
center=false, $fn=Curve_Points);
```

```
    difference(){
      Lid();
     //Next line generates a nitrogen inlet hole for lids with no SubaSeal only.
     if((Nitrogen_Needle==true)&&(Lid_Config=="Electrodes Only"))
rotate([0,0,90])translate([0.75*Internal_Radius_Reactor,0,0])cylinder(h=10*t_h2, r=0.75,
center=true, $fn=Curve_Points);

     //Creates single holes in the lid.
     translate([0,Suba_Offset,0]){
     if((Number_Electrode_Holes==1)&&(Electrode_Hole_Shape=="Circular"))

     cylinder(h=10*t_h2, r=Electrode_Hole_Size/2, center=true, $fn=Curve_Points);
     if((Number_Electrode_Holes==1)&&(Electrode_Hole_Shape=="Square"))
cube([Electrode_Hole_Size/2, Electrode_Hole_Size/2,10*t_h2], center=true);
     if((Number_Electrode_Holes==1)&&(Electrode_Hole_Shape=="Rectangle"))
cube([Rectangular_Electrode_X, Rectangular_Electrode_Y, 10*t_h2], center=true);
     if((Number_Electrode_Holes==1)&&(Electrode_Hole_Shape=="Electrasyn"))
cube([Electrasyn_Electrode_X, Electrasyn_Electrode_Y, 10*t_h2], center=true);
     }




     //Next block of code creates two cylindrical holes in the lid

     if((Number_Electrode_Holes==2)&&(Electrode_Hole_Shape=="Circular"))


{translate([(Electrode_Separation/2)+(Electrode_Hole_Size/2),Suba_Offset,0])cylinder(h=10*
t_h2, r=Electrode_Hole_Size/2, center=true, $fn=Curve_Points);
      translate([-(Electrode_Separation/2)-
(Electrode_Hole_Size/2),Suba_Offset,0])cylinder(h=10*t_h2, r=Electrode_Hole_Size/2,
center=true, $fn=Curve_Points);}

     //Next block of code creates two square holes in the lid

     if((Number_Electrode_Holes==2)&&(Electrode_Hole_Shape=="Square"))


{translate([(Electrode_Separation/2)+(Electrode_Hole_Size/2),Suba_Offset,0])cube([Electro
de_Hole_Size, Electrode_Hole_Size,10*t_h2], center=true);
      translate([-(Electrode_Separation/2)-
(Electrode_Hole_Size/2),Suba_Offset,0])cube([Electrode_Hole_Size,
Electrode_Hole_Size,10*t_h2], center=true);}

     //Next block of code creates two rectangular holes in the lid

     if((Number_Electrode_Holes==2)&&(Electrode_Hole_Shape=="Rectangle"))


{translate([(Electrode_Separation/2)+(Rectangular_Electrode_X/2),Suba_Offset,0])cube([Re
ctangular_Electrode_X, Rectangular_Electrode_Y, 10*t_h2], center=true);
```

```
        translate([-(Electrode_Separation/2)-
(Rectangular_Electrode_X/2),Suba_Offset,0])cube([Rectangular_Electrode_X,
Rectangular_Electrode_Y, 10*t_h2], center=true);}

    //Next block of code creates two Electrasyn electrode holes in the lid

        if((Number_Electrode_Holes==2)&&(Electrode_Hole_Shape=="Electrasyn"))


{translate([(Electrode_Separation/2)+(Electrasyn_Electrode_X/2),Suba_Offset,0])cube([Elect
rasyn_Electrode_X, Electrasyn_Electrode_Y, 10*t_h2], center=true);
        translate([-(Electrode_Separation/2)-
(Electrasyn_Electrode_X/2),Suba_Offset,0])cube([Electrasyn_Electrode_X,
Electrasyn_Electrode_Y, 10*t_h2], center=true);}

    //Next block of code creates three circular holes in a linear arrangement.

        if((Number_Electrode_Holes==3)&&(Electrode_Hole_Shape=="Circular"))


{translate([(Electrode_Separation)+(Electrode_Hole_Size),Suba_Offset,0])cylinder(h=10*t_h
2, r=Electrode_Hole_Size/2, center=true, $fn=Curve_Points);
        translate([-(Electrode_Separation)-
(Electrode_Hole_Size),Suba_Offset,0])cylinder(h=10*t_h2, r=Electrode_Hole_Size/2,
center=true, $fn=Curve_Points);
        translate([0,Suba_Offset,0])cylinder(h=10*t_h2, r=Electrode_Hole_Size/2, center=true,
$fn=Curve_Points);
            }

     //Next block of code creates three square holes in the lid in a linear arrangement.

     if((Number_Electrode_Holes==3)&&(Electrode_Hole_Shape=="Square"))


{translate([(Electrode_Separation)+(Electrode_Hole_Size),Suba_Offset,0])cube([Electrode_
Hole_Size, Electrode_Hole_Size,10*t_h2], center=true);
        translate([-(Electrode_Separation)-
(Electrode_Hole_Size),Suba_Offset,0])cube([Electrode_Hole_Size,
Electrode_Hole_Size,10*t_h2], center=true);
        translate([0,Suba_Offset,0])cube([Electrode_Hole_Size, Electrode_Hole_Size,10*t_h2],
center=true);
          }

     //Next block of code creates three rectangular holes in the lid in a linear arrangement.

     if((Number_Electrode_Holes==3)&&(Electrode_Hole_Shape=="Rectangle"))


{translate([(Electrode_Separation)+(Rectangular_Electrode_X),Suba_Offset,0])cube([Rectan
gular_Electrode_X, Rectangular_Electrode_Y, 10*t_h2], center=true);
        translate([-(Electrode_Separation)-
(Rectangular_Electrode_X),Suba_Offset,0])cube([Rectangular_Electrode_X,
Rectangular_Electrode_Y, 10*t_h2], center=true);
        translate([0,Suba_Offset,0])cube([Rectangular_Electrode_X, Rectangular_Electrode_Y,
10*t_h2], center=true);
```

```
        }

      //Next block of code creates three electrasyn electrode holes in the lid in a linear arrangement.

     if((Number_Electrode_Holes==3)&&(Electrode_Hole_Shape=="Electrasyn"))

{translate([(Electrode_Separation)+(Electrasyn_Electrode_X),Suba_Offset,0])cube([Electrasyn_Electrode_X, Electrasyn_Electrode_Y, 10*t_h2], center=true);
      translate([-(Electrode_Separation)-(Electrasyn_Electrode_X),Suba_Offset,0])cube([Electrasyn_Electrode_X, Electrasyn_Electrode_Y, 10*t_h2], center=true);
      translate([0,Suba_Offset,0])cube([Electrasyn_Electrode_X, Electrasyn_Electrode_Y, 10*t_h2], center=true);
        }

     //Next block of code creates three circular holes in a equidistant arrangement. (Suba-Seal not supported, limited space on reactor lid)

       if((Number_Electrode_Holes==4)&&(Electrode_Hole_Shape=="Circular"))
       {translate([0,Suba_Offset,0])

{translate([0,((0.5*Electrode_Separation)+(0.5*Electrode_Hole_Size))/cos(30),0])cylinder(h=10*t_h2, r=Electrode_Hole_Size/2, center=true, $fn=Curve_Points);

rotate([0,0,120])translate([0,((0.5*Electrode_Separation)+(0.5*Electrode_Hole_Size))/cos(30),0])cylinder(h=10*t_h2, r=Electrode_Hole_Size/2, center=true, $fn=Curve_Points);

rotate([0,0,240])translate([0,((0.5*Electrode_Separation)+(0.5*Electrode_Hole_Size))/cos(30),0])cylinder(h=10*t_h2, r=Electrode_Hole_Size/2, center=true, $fn=Curve_Points);
          }
       }
   //Next block of code creates three square holes in a equidistant arrangement. . (Suba-Seal not supported, limited space on reactor lid)

       if((Number_Electrode_Holes==4)&&(Electrode_Hole_Shape=="Square"))
        {translate([0,Suba_Offset,0])

{translate([0,((0.5*Electrode_Separation)+(0.5*Electrode_Hole_Size))/cos(30),0])cube([Electrode_Hole_Size, Electrode_Hole_Size,10*t_h2], center=true);

rotate([0,0,120])translate([0,((0.5*Electrode_Separation)+(0.5*Electrode_Hole_Size))/cos(30),0])cube([Electrode_Hole_Size, Electrode_Hole_Size,10*t_h2], center=true);

rotate([0,0,240])translate([0,((0.5*Electrode_Separation)+(0.5*Electrode_Hole_Size))/cos(30),0])cube([Electrode_Hole_Size, Electrode_Hole_Size,10*t_h2], center=true);      }
   }
   //Next block of code creates three rectangular holes in a equidistant arrangement.

       if((Number_Electrode_Holes==4)&&(Electrode_Hole_Shape=="Rectangle"))
       {translate([0,Suba_Offset,0])

{translate([0,((0.5*Electrode_Separation)+(0.5*Rectangular_Electrode_X))/cos(30),0])rotate([0,0,90])cube([Rectangular_Electrode_X, Rectangular_Electrode_Y, 10*t_h2], center=true);
```

```
rotate([0,0,120])translate([0,((0.5*Electrode_Separation)+(0.5*Rectangular_Electrode_X))/cos(30),0])rotate([0,0,90])cube([Rectangular_Electrode_X, Rectangular_Electrode_Y, 10*t_h2], center=true);

rotate([0,0,240])translate([0,((0.5*Electrode_Separation)+(0.5*Rectangular_Electrode_X))/cos(30),0])rotate([0,0,90])cube([Rectangular_Electrode_X, Rectangular_Electrode_Y, 10*t_h2], center=true);
          }
       }
    //Next block of code creates three Electrasyn holes in a equidistant arrangement. .

       if((Number_Electrode_Holes==4)&&(Electrode_Hole_Shape=="Electrasyn"))
       {translate([0,Suba_Offset,0])

{translate([0,((0.5*Electrode_Separation)+(0.5*Electrasyn_Electrode_X))/cos(30),0])rotate([0,0,90])cube([Electrasyn_Electrode_X, Electrasyn_Electrode_Y, 10*t_h2], center=true);

rotate([0,0,120])translate([0,((0.5*Electrode_Separation)+(0.5*Electrasyn_Electrode_X))/cos(30),0])rotate([0,0,90])cube([Electrasyn_Electrode_X, Electrasyn_Electrode_Y, 10*t_h2], center=true);

rotate([0,0,240])translate([0,((0.5*Electrode_Separation)+(0.5*Electrasyn_Electrode_X))/cos(30),0])rotate([0,0,90])cube([Electrasyn_Electrode_X, Electrasyn_Electrode_Y, 10*t_h2], center=true);
          }
       }

    //Next block creates 2 standard electrode holes, and adds a reference electrode close to the working electrode.

    if((Number_Electrode_Holes==5)&&(Electrode_Hole_Shape=="Circular"))

{translate([(Electrode_Separation/2)+(Electrode_Hole_Size/2),Suba_Offset+(0.5*Ref_Offset),0])cylinder(h=10*t_h2, r=Electrode_Hole_Size/2, center=true, $fn=Curve_Points);
      translate([-(Electrode_Separation/2)-(Electrode_Hole_Size/2),Suba_Offset+(0.5*Ref_Offset),0])cylinder(h=10*t_h2, r=Electrode_Hole_Size/2, center=true, $fn=Curve_Points);
       translate([-(Electrode_Separation/2)-(Electrode_Hole_Size/2),Suba_Offset-(0.5*Ref_Offset),0])cylinder(h=10*t_h2, r=Ref_Electrode_Diameter/2, center=true, $fn=Curve_Points);
          }

    //Next block of code creates two square holes in the lid + Reference

     if((Number_Electrode_Holes==5)&&(Electrode_Hole_Shape=="Square"))


{translate([(Electrode_Separation/2)+(Electrode_Hole_Size/2),Suba_Offset+(0.5*Ref_Offset),0])cube([Electrode_Hole_Size, Electrode_Hole_Size,10*t_h2], center=true);
      translate([-(Electrode_Separation/2)-(Electrode_Hole_Size/2),Suba_Offset+(0.5*Ref_Offset),0])cube([Electrode_Hole_Size, Electrode_Hole_Size,10*t_h2], center=true);
```

```
      translate([-(Electrode_Separation/2)-(Electrode_Hole_Size/2),Suba_Offset-(0.5*Ref_Offset),0])cylinder(h=10*t_h2, r=Ref_Electrode_Diameter/2, center=true, $fn=Curve_Points);
        }

    //This section creates Rectangle/Electrasyn Electrodes + Reference.

      if((Number_Electrode_Holes==5)&&(Electrode_Hole_Shape=="Rectangle"))


{translate([(Electrode_Separation/2)+(Rectangular_Electrode_X/2),Suba_Offset+(0.5*Ref_Offset_2),0])cube([Rectangular_Electrode_X, Rectangular_Electrode_Y, 10*t_h2], center=true);
      translate([-(Electrode_Separation/2)-(Rectangular_Electrode_X/2),Suba_Offset+(0.5*Ref_Offset_2),0])cube([Rectangular_Electrode_X, Rectangular_Electrode_Y, 10*t_h2], center=true);
      translate([-(Electrode_Separation/2)-(Rectangular_Electrode_X/2),Suba_Offset-(0.5*Ref_Offset_2),0])cylinder(h=10*t_h2, r=Ref_Electrode_Diameter/2, center=true, $fn=Curve_Points);
        }


    //Next block of code creates two Electrasyn electrode holes in the lid + Reference electrode.

      if((Number_Electrode_Holes==5)&&(Electrode_Hole_Shape=="Electrasyn"))


{translate([(Electrode_Separation/2)+(Electrasyn_Electrode_X/2),Suba_Offset+(0.5*Ref_Offset_3),0])cube([Electrasyn_Electrode_X, Electrasyn_Electrode_Y, 10*t_h2], center=true);
      translate([-(Electrode_Separation/2)-(Electrasyn_Electrode_X/2),Suba_Offset+(0.5*Ref_Offset_3),0])cube([Electrasyn_Electrode_X, Electrasyn_Electrode_Y, 10*t_h2], center=true);
      translate([-(Electrode_Separation/2)-(Rectangular_Electrode_X/2),Suba_Offset-(0.5*Ref_Offset_3),0])cylinder(h=10*t_h2, r=Ref_Electrode_Diameter/2, center=true, $fn=Curve_Points);
         }

           //Following section removes base of coil and suba channel


   if(Lid_Config=="Electrodes & Suba"){translate([0,0,-4*t_h2])cylinder(h=4*t_h2, r=Internal_Radius_Reactor+Wall_Thickness+t_d2, center=false);
    translate([0,-Internal_Radius_Reactor+Suba_Minor_Radius+2,0])cylinder(h=4*t_h2, r=Suba_Minor_Radius, center=false, $fn=Curve_Points);
   }
} //Closes difference.
if(Lid_Config=="Electrodes & Suba")translate([0,-Internal_Radius_Reactor+Suba_Minor_Radius+2,t_h2+2+3+5+1+1])Suba_Cone();
}
}

//Below module combines a Lid with Suba Cone and cuts the channel through the lid.

module Suba_Lid(){
```

```
    difference(){
       Lid();
       cylinder(h=4*t_h2, r=Suba_Minor_Radius, center=false, $fn=Curve_Points); //Cuts
channel
       translate([0,0,-4*t_h2])cylinder(h=4*t_h2,
r=Internal_Radius_Reactor+Wall_Thickness+t_d2, center=false); //Cuts off bottom of screw
thread to create flat base for printing
    }
    translate([0,0,t_h2+2+3+5+1+1])Suba_Cone();
}

//For Suba and electrodes, Suba is offset in Y as far as possible. Below is the limited case of
moving the position of the suba on the lid. Module not used in standard ERCAD options set.

module ES_Lid(){
    difference(){
       Lid();
       translate([0,Internal_Radius_Reactor-Suba_Minor_Radius-2,0])cylinder(h=4*t_h2,
r=Suba_Minor_Radius, center=false, $fn=Curve_Points); //Cuts channel
       translate([0,0,-4*t_h2])cylinder(h=4*t_h2,
r=Internal_Radius_Reactor+Wall_Thickness+t_d2, center=false); //Cuts off bottom of screw
thread to create flat base for printing
    }
    translate([0,Internal_Radius_Reactor-Suba_Minor_Radius-
2,t_h2+2+3+5+1+1])Suba_Cone();
}

module UndividedEsyn() {
      //Fixed diameter screw thread neck section

       translate([0,0,Height_Reactor-Base_Thickness-11]){

   difference(){
       union(){
          cylinder(r=t_d2_Esyn*0.5, h=11, center=false, $fn=Curve_Points);
          screw_thread(t_d1_Esyn, t_d2_Esyn, t_h1_Esyn, t_h2_Esyn+0.1);
          translate([0,0,0])cylinder(r=t_d2_Esyn*0.5, h=2, center=false, $fn=Curve_Points);
       }
     cylinder(h=4*t_h2_Esyn, r1=8.75, r2=8.75, center=true, $fn=Curve_Points);

       }
    }
    //Expansion Region to accomodate desired volume
       translate([0,0,Height_Reactor-Base_Thickness-10-11]){
          difference(){
             cylinder(10,r1=Internal_Radius_Esyn+Wall_Thickness,r2=0.5*t_d2_Esyn,
center=false, $fn=Curve_Points);
             cylinder(10,r1=Internal_Radius_Esyn, r2=8.75, center=false, $fn=Curve_Points);
             cylinder(0.1, r1=Internal_Radius_Esyn, r2=Internal_Radius_Esyn, center=true,
$fn=Curve_Points);
             translate([0,0,10])cylinder(0.1, r1=8.75, r2=8.75, center=true, $fn=Curve_Points);
          }
       }

    //Reactor main wall section
```

```
        difference(){
          cylinder(Height_Reactor-21-
Base_Thickness,r=Internal_Radius_Esyn+Wall_Thickness, center=false,
$fn=Curve_Points);
          cylinder(Height_Reactor-21+0.1-Base_Thickness, r=Internal_Radius_Esyn,
center=false, $fn=Curve_Points);
        }

    //Base Fillet
    Internal_Reactor_Fillet_Esyn();
    //Reactor base section
        translate([0,0,-Base_Thickness]){
        cylinder(h=Base_Thickness+0.01, r=Wall_Thickness+Internal_Radius_Esyn, center=
false, $fn=Curve_Points);
        }
    }

//Module below creates divided cells, is dependent on the Undivided Cell module for a basic
reactor body with screw thread.

module DividedA (){
    //This module uses difference to remove the required holes from a reactor unit constructed
as a union.
    difference(){
        union(){
          Undivided();

//Section below uses Hull to create a shape between a circle and rectangle, hollow out the
reactor volume with a difference, and then extrude vertically.
          translate([0,0,-
Base_Thickness])linear_extrude(Height_Reactor+Base_Thickness+0.01){
          difference(){
            hull(){
              circle(r=Wall_Thickness+Internal_Radius_Reactor);
              translate([Internal_Radius_Reactor,-0.5*Divider_Width,-Base_Thickness])
square([Wall_Thickness+Dividing_Wall_Thickness,Divider_Width], center=false);
                }
            circle(r=Internal_Radius_Reactor);
        }
    }
    //Section below creates the wings for bolting halves together
          translate([Internal_Radius_Reactor,-0.5*Divider_Width-Divider_Bolt_Section,-
Base_Thickness])cube([Wall_Thickness+Dividing_Wall_Thickness,Divider_Bolt_Section,Hei
ght_Reactor+Base_Thickness], center=false);
          translate([Internal_Radius_Reactor,0.5*Divider_Width,-
Base_Thickness])cube([Wall_Thickness+Dividing_Wall_Thickness,Divider_Bolt_Section,Hei
ght_Reactor+Base_Thickness], center=false);


        }
        //Next 6 lines creates bolt channels
        translate([Internal_Radius_Reactor-0.1,(0.5*Divider_Width)+(0.5*Divider_Bolt_Section),
0.15*Height_Reactor])rotate([0,90,0])cylinder(h=Wall_Thickness+Dividing_Wall_Thickness+
0.2,r=(0.5*Bolt_Size)+0.5, center=false, $fn=Curve_Points);
```

```
        translate([Internal_Radius_Reactor-0.1,(0.5*Divider_Width)+(0.5*Divider_Bolt_Section),
0.5*Height_Reactor])rotate([0,90,0])cylinder(h=Wall_Thickness+Dividing_Wall_Thickness+0.
2,r=(0.5*Bolt_Size)+0.5, center=false, $fn=Curve_Points);

        translate([Internal_Radius_Reactor-0.1,(0.5*Divider_Width)+(0.5*Divider_Bolt_Section),
0.85*Height_Reactor])rotate([0,90,0])cylinder(h=Wall_Thickness+Dividing_Wall_Thickness+
0.2,r=(0.5*Bolt_Size)+0.5, center=false, $fn=Curve_Points);

        translate([Internal_Radius_Reactor-0.1,-(0.5*Divider_Width)-
(0.5*Divider_Bolt_Section),
0.15*Height_Reactor])rotate([0,90,0])cylinder(h=Wall_Thickness+Dividing_Wall_Thickness+
0.2,r=(0.5*Bolt_Size)+0.5, center=false, $fn=Curve_Points);

        translate([Internal_Radius_Reactor-0.1,-(0.5*Divider_Width)-
(0.5*Divider_Bolt_Section),
0.5*Height_Reactor])rotate([0,90,0])cylinder(h=Wall_Thickness+Dividing_Wall_Thickness+0.
2,r=(0.5*Bolt_Size)+0.5, center=false, $fn=Curve_Points);

        translate([Internal_Radius_Reactor-0.1,-(0.5*Divider_Width)-
(0.5*Divider_Bolt_Section),
0.85*Height_Reactor])rotate([0,90,0])cylinder(h=Wall_Thickness+Dividing_Wall_Thickness+
0.2,r=(0.5*Bolt_Size)+0.5, center=false, $fn=Curve_Points);

       //Next line creates channel linking atmosphere of each compartment

        translate([Internal_Radius_Reactor-0.1,0,
0.85*Height_Reactor])rotate([0,90,0])cylinder(h=Wall_Thickness+Dividing_Wall_Thickness+
0.2,r=1, center=false, $fn=0.25*Curve_Points);

       //Next line creates channel which connects compartments (and will be covered by frit)
        translate([0.9*Internal_Radius_Reactor,0,
0.25*Height_Reactor])rotate([0,90,0])cylinder(h=2*(Wall_Thickness+Dividing_Wall_Thicknes
s),r=(0.25*Divider_Diameter), center=false, $fn=Curve_Points);
    }
}

//Module code for the Divided Cell Part B takes Part A and makes an extra space for the
Frit+O-ring to sit in.

module DividedB(){
    difference(){
        DividedA();
        translate([Internal_Radius_Reactor+Wall_Thickness+Dividing_Wall_Thickness-
Frit_Thickness+0.1,0,
0.25*Height_Reactor])rotate([0,90,0])cylinder(h=Frit_Thickness,r=(0.5*Divider_Diameter),
center=false, $fn=Curve_Points);
    }
}

module MultiUndivided(){
   for(i=[1:Number_Reactors]){
       rotate([0,0,i*(360/Number_Reactors)])translate([Translation_Distance,0,0]) Undivided();
       rotate([0,0,-
i*(360/Number_Reactors)])translate([Translation_Distance+Internal_Radius_Reactor+0.5*W
```

```
all_Thickness,0,Height_Reactor+0.75*t_h2])rotate([0,90,0])linear_extrude(0.75*Wall_Thickn
ess, center=false){
       text(str(i,"-",Reactor_Text), size=5, valign="center", halign="left");
      }
   }
}

module MultiUndivided_Support1(){
   for(i=[1:Number_Reactors]){
      rotate([0,0,i*(360/Number_Reactors)])translate([Translation_Distance,0,0])
cylinder(r=Internal_Radius_Reactor);
   }
}

module MultiUndivided_Support2(){
   translate([0,0,-Base_Thickness])linear_extrude(height=0.4*Height_Reactor){

       difference(){
          hull(){

             offset(delta=Wall_Thickness, chamfer=true){
                projection( cut=true) MultiUndivided_Support1();
                }}
          hull(){projection(cut=true) MultiUndivided_Support1();
                }
  }
       difference(){
          circle(r=Translation_Distance-Internal_Radius_Reactor);
          circle(r=Translation_Distance-Internal_Radius_Reactor-Wall_Thickness);
       }
  }

}


//Module positions multiple reactors based on the MultiDividedA_2 module below it.
module MultiDividedA(){
  for(i=[1:Number_Reactors]){
      rotate([0,0,i*(360/Number_Reactors)])translate([Translation_Distance,0,0])
MultiDividedA_2();
      rotate([0,0,-i*(360/Number_Reactors)])translate([Translation_Distance-
Internal_Radius_Reactor-
0.5*Wall_Thickness,0,Height_Reactor+0.75*t_h2])rotate([0,90,180])linear_extrude(0.75*Wall
_Thickness, center=false){
       text(str(i,"-",Reactor_Text), size=5, valign="center", halign="left");
      }
  }
}

//Module below constructs the divided cell, but makes adjustments to the face plate in order
to make low volume screening platforms possible with a single design.

module MultiDividedA_2 (){
   //This module uses difference to remove the required holes from a reactor unit constructed
as a union.
```

```
   difference(){
      union(){
         Undivided();

//Section below uses Hull to create a support shape between a circle and rectangle, hollow
out the reactor volume with a difference, and then extrude vertically.
         translate([0,0,-
Base_Thickness])linear_extrude(Height_Reactor+Base_Thickness+0.01){
         difference(){
            hull(){
               circle(r=Wall_Thickness+Internal_Radius_Reactor);
               translate([Internal_Radius_Reactor,-0.5*Divider_Width,-Base_Thickness])
square([Wall_Thickness+Dividing_Wall_Thickness,Divider_Width], center=false);
                  }
            circle(r=Internal_Radius_Reactor);
      }
   }

   //Section below creates the wings for bolting halves together
         translate([Internal_Radius_Reactor,-0.5*Divider_Width-Divider_Bolt_Section,-
Base_Thickness])cube([Wall_Thickness+Dividing_Wall_Thickness,Divider_Bolt_Section,Hei
ght_Reactor+Base_Thickness], center=false);
         translate([Internal_Radius_Reactor,0.5*Divider_Width,-
Base_Thickness])cube([Wall_Thickness+Dividing_Wall_Thickness,Divider_Bolt_Section,Hei
ght_Reactor+Base_Thickness], center=false);


      }
      //Next 6 lines creates bolt channels
      translate([Internal_Radius_Reactor-0.1,(0.5*Divider_Width)+(0.5*Divider_Bolt_Section),
0.15*Height_Reactor])rotate([0,90,0])cylinder(h=Wall_Thickness+Dividing_Wall_Thickness+
0.2,r=(0.5*Bolt_Size)+0.5, center=false, $fn=Curve_Points);

      translate([Internal_Radius_Reactor-0.1,(0.5*Divider_Width)+(0.5*Divider_Bolt_Section),
0.5*Height_Reactor])rotate([0,90,0])cylinder(h=Wall_Thickness+Dividing_Wall_Thickness+0.
2,r=(0.5*Bolt_Size)+0.5, center=false, $fn=Curve_Points);

      translate([Internal_Radius_Reactor-0.1,(0.5*Divider_Width)+(0.5*Divider_Bolt_Section),
0.85*Height_Reactor])rotate([0,90,0])cylinder(h=Wall_Thickness+Dividing_Wall_Thickness+
0.2,r=(0.5*Bolt_Size)+0.5, center=false, $fn=Curve_Points);

      translate([Internal_Radius_Reactor-0.1,-(0.5*Divider_Width)-
(0.5*Divider_Bolt_Section),
0.15*Height_Reactor])rotate([0,90,0])cylinder(h=Wall_Thickness+Dividing_Wall_Thickness+
0.2,r=(0.5*Bolt_Size)+0.5, center=false, $fn=Curve_Points);

      translate([Internal_Radius_Reactor-0.1,-(0.5*Divider_Width)-
(0.5*Divider_Bolt_Section),
0.5*Height_Reactor])rotate([0,90,0])cylinder(h=Wall_Thickness+Dividing_Wall_Thickness+0.
2,r=(0.5*Bolt_Size)+0.5, center=false, $fn=Curve_Points);

      translate([Internal_Radius_Reactor-0.1,-(0.5*Divider_Width)-
(0.5*Divider_Bolt_Section),
0.85*Height_Reactor])rotate([0,90,0])cylinder(h=Wall_Thickness+Dividing_Wall_Thickness+
0.2,r=(0.5*Bolt_Size)+0.5, center=false, $fn=Curve_Points);
```

```
      //Next line creates channel linking atmosphere of each compartment

       translate([Internal_Radius_Reactor-0.1,0,
0.85*Height_Reactor])rotate([0,90,0])cylinder(h=Wall_Thickness+Dividing_Wall_Thickness+
0.2,r=1, center=false, $fn=0.25*Curve_Points);

      //Next line creates channel which connects compartments (and will be covered by frit)
       translate([0.9*Internal_Radius_Reactor,0,
0.25*Height_Reactor])rotate([0,90,0])cylinder(h=2*(Wall_Thickness+Dividing_Wall_Thicknes
s),r=(0.25*Divider_Diameter), center=false, $fn=Curve_Points);
   }
}



//Below module draws rectangles on the inner edge of the dividing wall, which are then
joined with hull to give an outline of the footprint of the reactor (with offset to create the
width, see Support2). However, when these rectangles overlap (mainly with large number of
small reactors with big bolts) the rectangles overlap, and therefore the hull operation makes
the support material further away from the reactor than it should be.
module MultiDivided_Support1(){

   for(i=[1:Number_Reactors]){

rotate([0,0,i*(360/Number_Reactors)])translate([Translation_Distance+Internal_Radius_Rea
ctor,0,0]) square([0.1,Divider_Width+2*Divider_Bolt_Section], center=true);
  }
}


//Make support and then use difference to remove cylinders needed from it.
module MultiDivided_Support2(){
  difference(){
      translate([0,0,-Base_Thickness])linear_extrude(height=0.4*Height_Reactor){

         difference(){
            hull(){

               offset(r=Dividing_Wall_Thickness+Wall_Thickness, chamfer=true){
               MultiDivided_Support1();
               }}
            hull() MultiDivided_Support1();
               }

         difference(){
            circle(r=Translation_Distance-Internal_Radius_Reactor);
            circle(r=Translation_Distance-Internal_Radius_Reactor-Wall_Thickness);
            }
       }
      for(i=[1:Number_Reactors]){

         //Remove frit area

rotate([0,0,i*(360/Number_Reactors)])translate([Translation_Distance+0.5*Internal_Radius_
```

```
Reactor,0,
0.25*Height_Reactor])rotate([0,90,0])cylinder(h=Internal_Radius_Reactor+2*(Wall_Thicknes
s+Dividing_Wall_Thickness),r=(0.25*Divider_Diameter), center=false, $fn=Curve_Points);

        //Remove bolt channels

rotate([0,0,i*(360/Number_Reactors)])translate([Translation_Distance+Internal_Radius_Rea
ctor-0.1,(0.5*Divider_Width)+(0.5*Divider_Bolt_Section),
0.15*Height_Reactor])rotate([0,90,0])cylinder(h=4*Wall_Thickness+Dividing_Wall_Thickness
+0.2,r=(0.5*Bolt_Size)+0.5, center=false, $fn=Curve_Points);

rotate([0,0,i*(360/Number_Reactors)])translate([Translation_Distance+Internal_Radius_Rea
ctor-0.1,-(0.5*Divider_Width)-(0.5*Divider_Bolt_Section),
0.15*Height_Reactor])rotate([0,90,0])cylinder(h=4*Wall_Thickness+Dividing_Wall_Thickness
+0.2,r=(0.5*Bolt_Size)+0.5, center=false, $fn=Curve_Points);
      }
  }
}


/*---------------------------------------------------
TOP LINE OBJECTS TO RENDER */

// Undivided Electrasyn Single Reactor

if((Part=="Undivided
Reactor")&&(Compartments=="Undivided")&&(Electrasyn_Compatibility==true)&&(Number_
Reactors==1)) {UndividedEsyn();

translate([Internal_Radius_Esyn+0.5*Wall_Thickness,0,0.5*Height_Reactor])rotate([0,90,0])l
inear_extrude(0.75*Wall_Thickness, center=false){
      text(str(Reactor_Text), size=5, valign="center");
   }
}

// Undivided Non-Electrasyn Single Reactor
if((Part=="Undivided
Reactor")&&(Electrasyn_Compatibility==false)&&(Compartments=="Undivided")&&(Number
_Reactors==1)){ Undivided();

translate([Internal_Radius_Reactor+0.5*Wall_Thickness,0,Height_Reactor+0.75*t_h2])rotate
([0,90,0])linear_extrude(0.75*Wall_Thickness, center=false){
      text(str(Reactor_Text), size=5, valign="center", halign="left");
   }
}

//Single Divided Cell Reactor Part A
if((Part=="Divided Part
A")&&(Compartments=="Divided")&&(Number_Reactors==1)&&(Electrasyn_Compatibility==f
alse)){ DividedA();
translate([-Internal_Radius_Reactor-
0.5*Wall_Thickness,0,Height_Reactor+0.75*t_h2])rotate([0,90,180])linear_extrude(0.75*Wall
_Thickness, center=false){
      text(str(Reactor_Text), size=5, valign="center", halign="left");
   }
```

```
}

//Divided Cell Part B (Same for screening or standalone reactor)
if((Part=="Divided Part
B")&&(Compartments=="Divided")&&(Electrasyn_Compatibility==false)){ DividedB();

translate([-Internal_Radius_Reactor-
0.5*Wall_Thickness,0,Height_Reactor+0.75*t_h2])rotate([0,90,180])linear_extrude(0.75*Wall
_Thickness, center=false){
      text(str(Reactor_Text), size=5, valign="center", halign="left");
   }
}

//Screw fit Lids (Same for Divided or undivided), different configuration options.
if((Part=="Lid")&&(Lid_Config=="Electrodes Only")) Electrode_Lid();

if((Part=="Lid")&&(Lid_Config=="Electrodes & Suba")) Electrode_Lid();

if((Part=="Lid")&&(Lid_Config=="Suba Only")) Suba_Lid();


// Screening Array of Undivided Cells
if((Part=="Undivided
Reactor")&&(Compartments=="Undivided")&&(Electrasyn_Compatibility==false)&&(Number
_Reactors!=1)){ MultiUndivided();
   MultiUndivided_Support2();
}

//Screening Array of Divided Cells.

if((Part=="Divided Part
A")&&(Compartments=="Divided")&&(Electrasyn_Compatibility==false)&&(Number_Reactor
s!=1)){
   MultiDividedA();
   MultiDivided_Support2();
}
```

Reactor Presets


```
{
   "parameterSets": {
      "N-N Dimer": {
         "Base_Curvature": "50",
         "Base_Thickness": "3",
         "Bolt_Size": "4",
         "Compartments": "Undivided",
         "Curve_Points": "20",
         "Divider_Diameter": "20",
         "Dividing_Wall_Thickness": "2",
         "Electrasyn_Compatibility": "false",
         "Electrasyn_Electrode_X": "2.2",
         "Electrasyn_Electrode_Y": "9",
         "Electrode_Hole_Shape": "Electrasyn",
         "Electrode_Hole_Size": "5",
```

"Electrode_Separation": "5",
"Frit_Thickness": "2",
"HR_Ratio": "4",
"Label": "N-N Synth",
"Lid_Config": "Electrodes Only",
"Nitrogen_Needle": "false",
"Number_Electrode_Holes": "2",
"Number_Reactors": "1",
"Part": "Undivided Reactor",
"Part_Quality": "1",
"Push_Fit": "false",
"Rectangular_Electrode_X": "1",
"Rectangular_Electrode_Y": "3",
"Ref_Electrode": "false",
"Ref_Electrode_Diameter": "6",
"Ref_Separation": "5",
"Suba_Size": "12",
"VolumeML": "8",
"Wall_Thickness": "3",
"fn_value": "16",
"t_d1_Esyn": "2.36",
"t_d2_Esyn": "22.5",
"t_h1": "1.14",
"t_h1_Esyn": "1.14",
"t_h2": "10",
"t_h2_Esyn": "10"
},
"Acetoxylation": {
"Base_Curvature": "50",
"Base_Thickness": "3",
"Bolt_Size": "4",
"Compartments": "Undivided",
"Curve_Points": "20",
"Divider_Diameter": "20",
"Dividing_Wall_Thickness": "2",
"Electrasyn_Compatibility": "false",
"Electrasyn_Electrode_X": "2.2",
"Electrasyn_Electrode_Y": "9",
"Electrode_Hole_Shape": "Electrasyn",
"Electrode_Hole_Size": "5",
"Electrode_Separation": "2",
"Frit_Thickness": "2",
"HR_Ratio": "4",
"Label": "Acetoxylation",
"Lid_Config": "Electrodes Only",
"Nitrogen_Needle": "true",
"Number_Electrode_Holes": "2",
"Number_Reactors": "1",
"Part": "Lid",
"Part_Quality": "2",
"Push_Fit": "false",
"Rectangular_Electrode_X": "1",
"Rectangular_Electrode_Y": "3",
"Ref_Electrode": "false",
"Ref_Electrode_Diameter": "6",

"Ref_Separation": "5",
"Suba_Size": "12",
"VolumeML": "6",
"Wall_Thickness": "3",
"fn_value": "16",
"t_d1_Esyn": "2.36",
"t_d2_Esyn": "22.5",
"t_h1": "1.14",
"t_h1_Esyn": "1.14",
"t_h2": "10",
"t_h2_Esyn": "10"
},
"CV Cell v2": {
"Base_Curvature": "25",
"Base_Thickness": "2",
"Bolt_Size": "4",
"Compartments": "Undivided",
"Curve_Points": "20",
"Divider_Diameter": "20",
"Dividing_Wall_Thickness": "2",
"Electrasyn_Compatibility": "false",
"Electrasyn_Electrode_X": "2.2",
"Electrasyn_Electrode_Y": "9",
"Electrode_Hole_Shape": "Circular",
"Electrode_Hole_Size": "6.5",
"Electrode_Separation": "1.5",
"Frit_Thickness": "2",
"HR_Ratio": "4",
"Label": "CV Cell",
"Lid_Config": "Electrodes Only",
"Nitrogen_Needle": "true",
"Number_Electrode_Holes": "5",
"Number_Reactors": "1",
"Part": "Lid",
"Part_Quality": "3",
"Push_Fit": "false",
"Rectangular_Electrode_X": "1",
"Rectangular_Electrode_Y": "3",
"Ref_Electrode": "false",
"Ref_Electrode_Diameter": "3.5",
"Ref_Separation": "1.5",
"Suba_Size": "12",
"VolumeML": "8",
"Wall_Thickness": "2",
"fn_value": "16",
"t_d1_Esyn": "2.36",
"t_d2_Esyn": "22.5",
"t_h1": "1.14",
"t_h1_Esyn": "1.14",
"t_h2": "10",
"t_h2_Esyn": "10"
},
"40 mL Acetoxylation": {
"Base_Curvature": "20",
"Base_Thickness": "2",

"Bolt_Size": "4",
"Compartments": "Undivided",
"Divider_Diameter": "20",
"Dividing_Wall_Thickness": "2",
"Electrasyn_Compatibility": "true",
"Electrasyn_Electrode_X": "2.2",
"Electrasyn_Electrode_Y": "9",
"Electrode_Hole_Shape": "Circular",
"Electrode_Hole_Size": "6.3",
"Electrode_Separation": "3",
"Frit_Thickness": "2",
"HR_Ratio": "4",
"Label": "LennoxLab",
"Lid_Config": "Electrodes Only",
"Lid_Seal": "false",
"Nitrogen_Needle": "true",
"Number_Electrode_Holes": "2",
"Number_Reactors": "1",
"Part": "Undivided Reactor",
"Part_Quality": "3",
"Push_Fit": "false",
"Rectangular_Electrode_X": "1",
"Rectangular_Electrode_Y": "3",
"Ref_Electrode": "false",
"Ref_Electrode_Diameter": "6",
"Ref_Separation": "5",
"Seal_Thickness": "4",
"Suba_Size": "12",
"VolumeML": "40",
"Wall_Thickness": "2",
"fn_value": "16",
"t_d1_Esyn": "2.36",
"t_d2_Esyn": "22.5",
"t_h1": "1.14",
"t_h1_Esyn": "1.14",
"t_h2": "10",
"t_h2_Esyn": "10"
}
},
"fileFormatVersion": "1",
"parameterSets": "",
"parameterSets": ""
}

References

[1] W. C. Still, M. Kahn, A. Mitra, *J. Org. Chem.* **1978**, *43*, 2923–2925.

[2] S. Doobary, A. T. Sedikides, H. P. Caldora, D. L. Poole, A. J. J. Lennox, *Angew. Chem. Int. Ed.* **2020**, *59*, 1155–1160.

[3] P. J. Kitson, S. Glatzel, W. Chen, C. G. Lin, Y. F. Song, L. Cronin, *Nat Protoc* **2016**, *11*, 920–936.

[4] "The IKA Electrasyn Pro-Divide (https://www.ika.com/en/Products-Lab-Eq/Electrochemistry-Kit-csp-516/IKA-Pro-Divide-cpdt-40006482/) is a commercial solution to performing divided cell chemistry on the Electrasyn platform.," **n.d.**

[5] A. P. Atkins, A. C. Rowett, D. M. Heard, J. A. Tate, A. J. J. Lennox, *Org. Lett.* **2022**, *24*, 5105–5108.

[6] T. Gieshoff, D. Schollmeyer, S. R. Waldvogel, *Angew. Chem. Int. Ed.* **2016**, *55*, 9437–9440.
